# Infants have rich visual categories in ventrotemporal cortex at 2 months of age

**DOI:** 10.1038/s41593-025-02187-8

**Published:** 2026-02-02

**Authors:** Cliona O’Doherty, Áine T. Dineen, Anna Truzzi, Graham King, Lorijn Zaadnoordijk, Keelin Harrison, Enna-Louise D’Arcy, Jessica White, Chiara Caldinelli, Tamrin Holloway, Anna Kravchenko, Jörn Diedrichsen, Ailbhe Tarrant, Angela T. Byrne, Adrienne Foran, Eleanor J. Molloy, Rhodri Cusack

**Affiliations:** 1 Trinity College Institute of Neuroscience, Trinity College Dublin, Dublin, Ireland; 2 School of Psychology, Trinity College Dublin, Dublin, Ireland; 3 School of Psychology, Queen’s University Belfast, Belfast, UK; 4 Center for Brain and Cognition, DTIC, Universitat Pompeu Fabra, Barcelona, Spain; 5 Helen Wills Neuroscience Institute & Department of Psychology, University of California, Berkeley, Berkeley, CA USA; 6 Departments of Statistical and Actuarial Sciences and Computer Science, Western University, London, Ontario Canada; 7 Western Institute of Neuroscience, Western University, London, Ontario Canada; 8 The Rotunda Hospital, Dublin, Ireland; 9 Children’s Health Ireland (CHI), Dublin, Ireland; 10 Coombe Women’s and Infant’s University Hospital, Dublin, Ireland; 11 Discipline of Paediatrics, Trinity College Dublin, Dublin, Ireland; 12 Trinity Translational Medicine Institute (TTMI), St. James Hospital & Trinity Research in Childhood Centre (TRiCC), Dublin, Ireland

**Keywords:** Cognitive neuroscience, Network models, Development of the nervous system, Object vision

## Abstract

What are the foundations of visual categories in the human brain? Although infant looking behavior characterizes the development of overt categorization, it cannot measure neural representation or distinguish the underlying mechanism. For this, we need rich neuroimaging from young infants and the capacity to apply advanced computational models of vision. In this study, we conducted an awake functional magnetic resonance imaging (fMRI) study of more than 100 2-month-old infants, with follow-ups at 9 months, finding that categorical structure is present in high-level visual cortex from 2 months of age. This precedes its emergence in lateral visual cortex, suggesting non-hierarchical development of category representations. A deep neural network model aligned with infants’ representational geometry, indicating that the features comprising infants’ category template span a range of complexities and can be learned from the statistics of visual input. Our results reveal the existence of complex function in ventral visual cortex at 2 months of age and describe the early development of category perception.

## Abstract (web summary)

Using infant fMRI, the authors show that, by 2 months of age, representations in high-level visual cortex encode visual categories that align with deep neural networks, and lateral object-selective regions are later to develop.

## Main

Infants must lay the foundations for cognition in the first year of life, with visual recognition being an important developmental challenge^1^. During this time, humans learn to recognize the things they see, grouping them into meaningful categories that later converge with language. Although understanding of visual processing in adults has made substantial progress^2–5^, its developmental trajectory remains unclear. Theories of visual development differ in emphasis on sensory-driven, bottom-up statistical learning^6^, the role of experience-dependent input^7,8^ or core knowledge systems^9^, hierarchical development from a primitive cortical organization^10^ and the degree of continuity between infants and adults^11,12^. However, brain development is likely a complex interplay between many of these factors^13,14^, and each element’s relative influence is difficult to distinguish without a characterization of visual function in early life.

The emergence of visual categories has been assessed in infants, with experimental paradigms measuring looking behavior. Young infants have been found to be sensitive to global structure and perceptual features^15,16^, and 10-month-old infants are sensitive to basic-level categories and conceptual features^17^. However, knowing when these functions appear in behavior does not reveal the developmental processes that led to their emergence. To distinguish these mechanisms, it would be valuable to characterize the precursor states of each element in the system. This would reveal how things change across time, through learning or maturation, and how brain function culminates in behavior. We aimed to do this by longitudinally measuring brain development, with a focus on the ventral visual stream (VVS). In adults, this region is key to visual recognition and comprises brain regions structured as a processing hierarchy from perceptual features to semantic categories^18,19^. In the visual system of infants, electroencephalography and magnetoencephalography (EEG and MEG) have found a staggered development of categories^20,21^ that mirrors the transition in feature complexity found in behavioral studies. However, the limited spatial resolution of these methods did not allow separation of the different parts of the ventral hierarchy. Methodological advances in awake infant fMRI have the potential to overcome this limitation^22,23^.

To characterize the developmental mechanisms, we related longitudinal fMRI measurements of regions along the VVS to computational models of vision. Deep neural networks (DNNs) are now cemented as effective models of the adult brain in several domains^24,25^, acting as complex feature detectors that learn the statistical regularities in visual input to encode an increasingly complex range of multidimensional features in their hierarchical layers—similar to adult visual cortex^26–28^. Particular progress has been made using representational similarity analysis (RSA)^29^, where the distributed codes for diverse stimuli are compared between the brain and the DNN to characterize the features encoded in each visual region. We presented a broader range of stimuli, avoiding those for which humans likely have specialized mechanisms, such as faces. This permits tests of alignment with computational models to determine if DNNs can be effective models in developmental neuroscience and to allow for discrimination of feature processing along the visual hierarchy.

We acquired a large-scale longitudinal MRI dataset of functional brain activity from awake infants, bridging the gap between large studies of sleeping or sedated infants^30–32^ and pioneering studies of awake infants with samples that collapse across different ages^22,23,33^. We found that a range of features are processed along the ventral hierarchy, including categorical representations across the ventral surface from 2 months of age. These representations corresponded to those in DNNs, revealing that the features encoded are like those that facilitate efficient categorization in machines. High-level vision continues to mature throughout the first year of life as the ventrotemporal cortex becomes more specialized in its stage-like function. We found later development of category representations in the object-selective lateral occipitotemporal cortex (LO), a region upstream of the anterior VVS in high-level visual processing^34,35^, revealing that human visual categories do not develop in a bottom-up manner along the cortical processing hierarchy.

## Infant visual representations measured with awake fMRI at scale

**Fig. 1:**
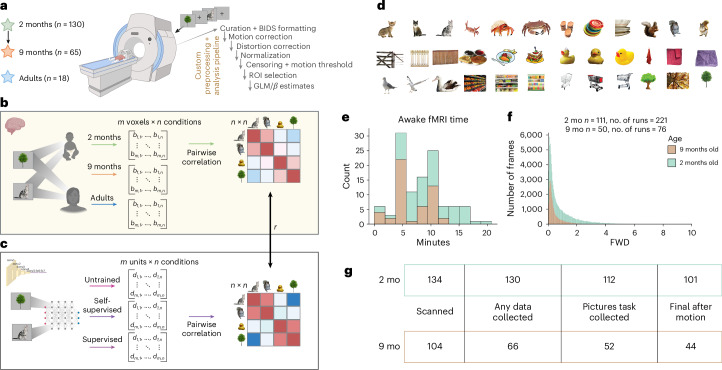
Characterizing the development of visual representations. **a**, Awake longitudinal fMRI was collected in 2-month-old infants and again at 9 months of age. A cohort of adults was collected for comparison. After careful data curation and preprocessing with custom analysis pipelines, response patterns were estimated using a GLM. The final dataset for the pictures task presented here, after motion thresholding, included *n* = 101 2-month-old infants and *n* = 44 9-month-old infants. Created in BioRender: O’doherty, C. (2025): https://BioRender.com/fyk6ao9. **b**, Visual representations were characterized per ROI and age group using multivariate pattern analysis. The pairwise correlations, across subjects and runs, between all *n* conditions *β* estimates in an *m-*voxel dimensional space gave an *n* *×* *n* similarity matrix. **c**, The equivalent analysis was performed in a DNN. Activations were calculated from each layer, in response to the same images used during scanning. The resulting model similarity matrix could then be compared with the fMRI data using a Spearman correlation. **d**, Stimuli used in the pictures task. Three exemplars were chosen per 12 categories spanning animate, inanimate-small and inanimate-large. **e**, Frequency of time spent in the awake fMRI pictures task for 2-month-old infants and 9-month-old infants. **f**, Histogram of FWD across all data collected. Frames with FWD > 1.5 mm were removed when fitting the GLM, and runs with more than half the frames above this threshold were not included in analyses. **g**, Number of participants collected at each stage of the study. Infants also completed an awake videos task, which will be released with future work. mo, months old. Source data

**Fig. 2:**
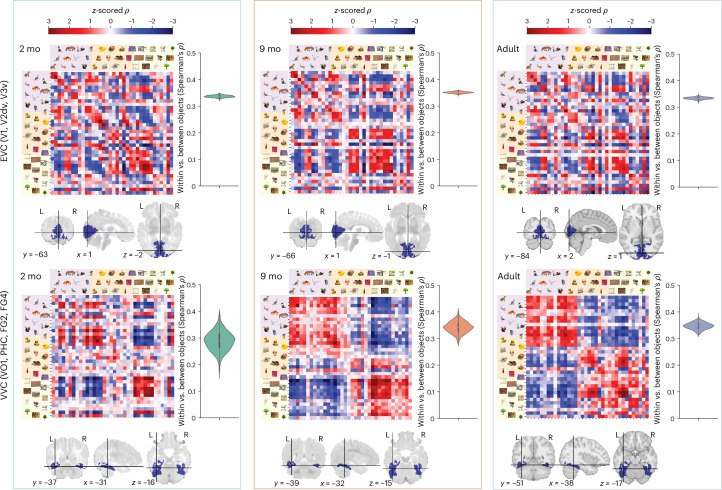
Visual representations from infancy to adulthood. The group average, across-subject pairwise correlation distance between voxelwise patterns from EVC and VVC in response to each object. Aggregated ROIs were defined using individual regions from the Julich atlas that have been validated to overlap with functionally defined regions. Conditions within the RSMs are nested by animacy class (animate, inanimate-small and inanimate-large), then category (four per animacy class) and, finally, exemplar (three per category). To focus on representational content rather than strength, we plotted the *z*-scored correlation distance. Raw group mean correlation ranges varied with age: 2-month EVC = (−0.027 to 0.030) and VVC = (−0.0082 to 0.0078); 9-month EVC = (−0.054 to 0.073) and VVC = (−0.030 to 0.045); adult EVC = (−0.112 to 0.142) and VVC = (−0.046 to 0.068). Violin plots show the bootstrap distribution of RSM correlation to a model that codes for within-object versus between-object similarity, implemented as an identity matrix. The violin is a symmetric kernel density estimate of the bootstrap distribution across all subject/run pair RSMs (2 months (*n* = 101): 14,108 unique subject/run pairs, 9 months (*n* = 44): 1,995 unique subject/run pairs; adults (*n* = 17): 136 unique subject/run pairs). The white dot is the median; the black bar is the IQR; and the whiskers denote 1.5 × IQR of the lower and upper quantiles. Correlations were normalized by the split-half noise ceiling for each ROI/age. ROIs are plotted from the atlas transformed into age-specific templates. mo, months old. Source data

**Extended Data Fig. 1:**
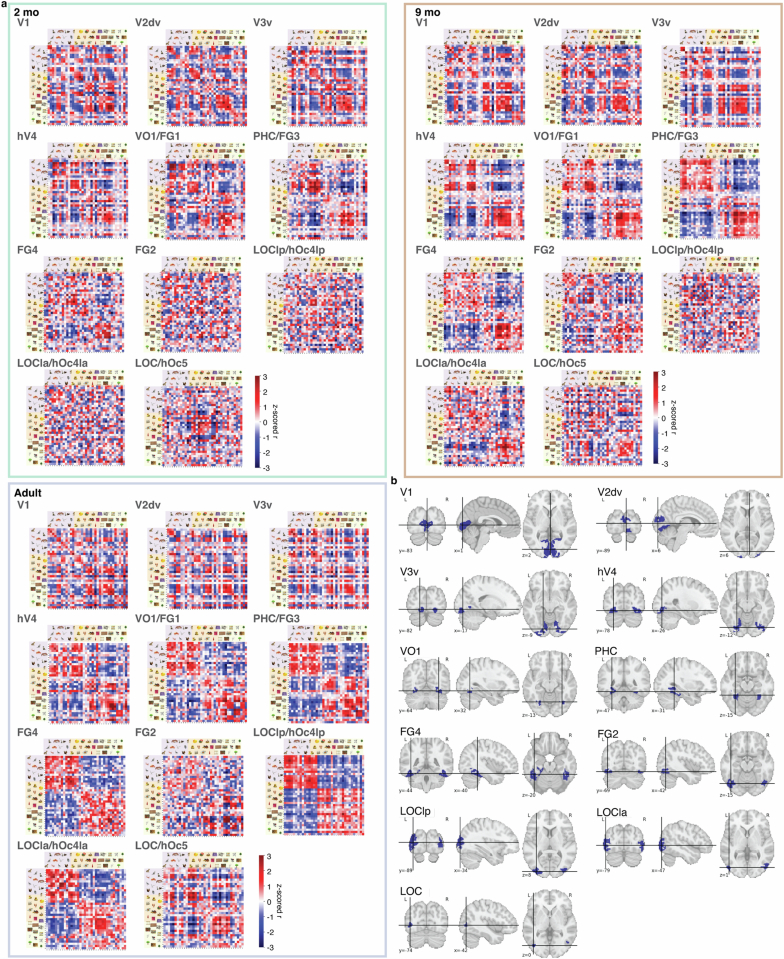
RSMs for all individual ROIs. **(a)** The group average, across subject pairwise correlation voxelwise responses patterns from all Julich-based ROIs. Conditions are nested by animacy, then category, and exemplar. To focus on representational content rather than strength, we plot the z-score of the correlation. **(b)** Individual ROIs in adult space. In practice, RSMs were calculated from the atlas transformed into an age specific template. Source data

fMRI was acquired longitudinally from 2-month-old (*n* = 130) and 9-month-old (*n* = 65) infants as they viewed 12 common visual categories (Fig. 1a–d). These were chosen across animate, small inanimate and large inanimate classes that would typically be seen in the first year of life (in person or through books), corresponding to words with a young age of acquisition^36^. For each category, we included three representative exemplars from diverse viewpoints to decorrelate perceptual and semantic, defined by category, responses. The success of awake fMRI depends upon infants staying content and relatively still in the scanner, so the pictures loomed to captivate attention. Most infants (63%) participated for at least four repetitions of each stimulus, totaling 144 pictures across 10 minutes of scanning. The distribution of head motion was acceptable (runs with a median framewise displacement (FWD) of <1.5 mm at 2 months, 85%; at 9 months, 97%) (Fig. 1d,f) and allowed for rigorous scrubbing during fMRI analysis (Methods). This resulted in a final sample of *n* = 101 2-month-old infants and *n* = 44 9-month-old infants for the pictures task. A cohort of adults (*n* = 17) was acquired for comparison. The blood oxygen level-dependent (BOLD) response to each object exemplar was estimated with a general linear model (GLM), and RSA was used to measure the representational geometry for each region in the ventral visual cortex (VVC). Individual regions of interest (ROIs) were defined using the cytoarchitectonic Julich atlas, which has been validated in its overlap with functionally defined regions^34^. To illustrate the broad picture, we first show early and late stages of processing the ventral stream (early visual cortex (EVC): comprising V1, V2dv and V3v; VVC: comprising FG1/VO1, FG3/PHC, FG2 and FG4). The similarities of voxelwise response patterns to each pair of conditions were calculated across subjects, giving a representational similarity matrix (RSM) for each ROI and age group (Fig. 2). The group mean RSMs were reliable, as estimated by a split-half noise ceiling, adjusting for test length using the Spearman−Brown correction (2 months of age: *ρ*(51) = 0.955 in EVC and 0.741 in VVC; 9 months of age: *ρ*(22) = 0.947 in EVC and 0.877 in VVC; adult: *ρ*(9) = 0.967 in EVC and 0.907 in VVC). Noise ceilings for all ROIs are reported in Extended Data Table 1, RSMs in Extended Data Fig. 1a–c and ROIs in Extended Data Fig. 1d. Correlations within each across-subject RSM ranged from (−1.0 to 1.0) for 2-month-old infants, from (−0.74 to 0.64) for 9-month-old infants and from (−0.32 to 0.42) for adults. To focus on signals that were highly consistent across participants, we report group mean RSMs across pairs of subjects.

## Basic and global categories are present in the ventral stream from 2 months of age

**Extended Data Fig. 2:**
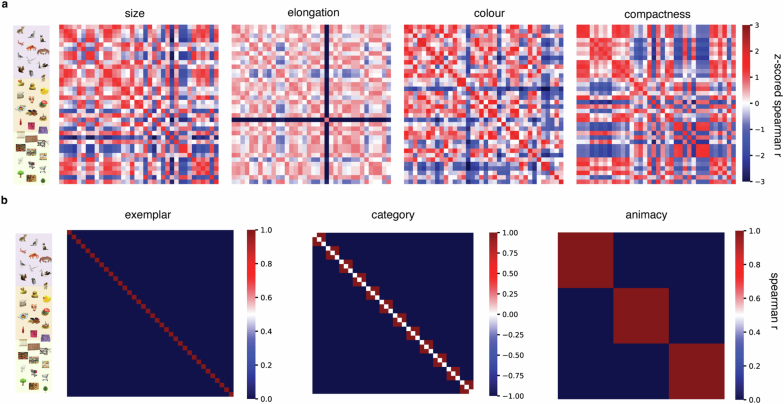
Perceptual and semantic model RSMS. **(a)** Perceptual RSMs for the features of size, elongation, color and compactness. The raw measurements of each feature were calculated for the 36 image stimuli, and 1 - pairwise distances entered as the distance value in the RSM. We plot the z-scored distances to aid comparison, as the raw values vary with feature. **(b)** Semantic feature RSMs. Raw values are plot to illustrate model definitions, but the appropriately weighted and normalised values were used when comparing to brain data. Source data

**Extended Data Fig. 3:**
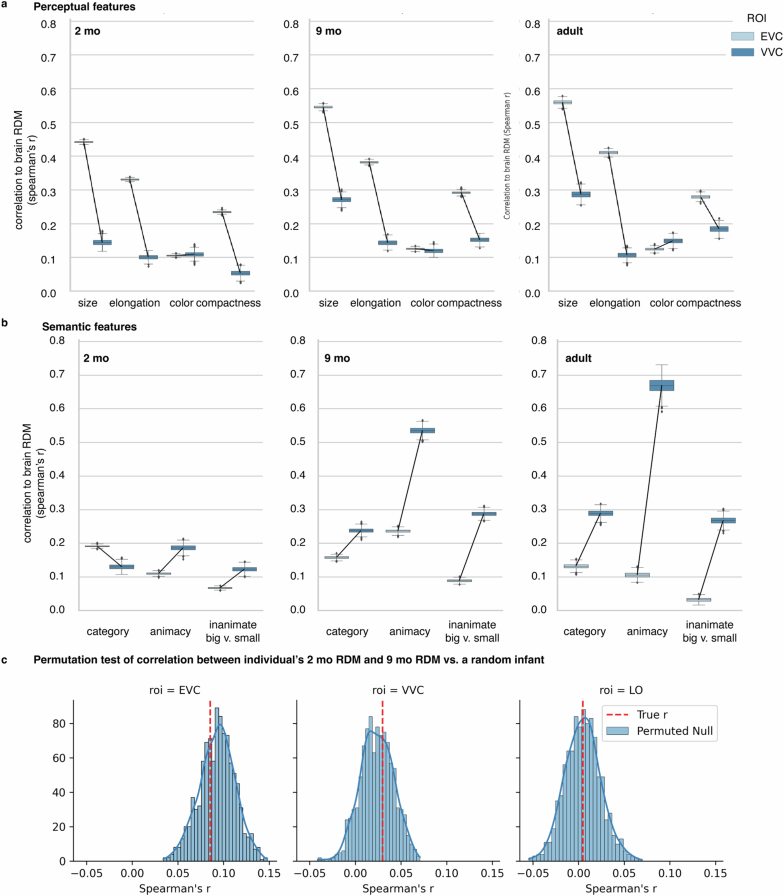
RSA replication in a motion matched subsample and longitudinal effects. **(a,b)** RSA results in a subsample of participants with matching motion distributions to the adult cohort, excluding the NICU group. Correlations are normalised by the motion-corrected noise ceiling per ROI/age-group. Boxes bounds are the 25^th^ and 75^th^ quartiles of the bootstrapped 95% confidence interval across subject pairs (2-months (n = 101): 14108 unique subject/run pairs, 9-months (n = 44): 1995 pairs, adults (n = 17): 136 pairs). The middle line is the median, whiskers extend to 1.5 x IQR, individual points are outliers **(c)** The average within subject correlation across 2 and 9 months (n = 38) is depicted in the red dashed line. This was compared to a null distribution, estimated using 1000 permutations shuffling across subjects and ensuring the same subject was not being correlated with themselves in the permuted test. We find no effect of longitudinal similarity in the representations of EVC (p = 0.656), VVC (p = 0.378) and LO (p = 0.507). Source data

Representations in both EVC and VVC were surprisingly mature by 2 months of age (correlation to adult group EVC *ρ* = 0.788, 95% bootstrap confidence interval (CI): 0.782−0.793; VVC *ρ* = 0.577, 95% CI: 0.558−0.598) with continued maturation throughout the first year of life (9-month correlation to adult EVC *ρ* = 0.792, 95% CI: 0.783−0.802; VVC *ρ* = 0.654, 95% CI: 0.634−0.670). All statistics, including the across-group differences and their significance, were calculated using bootstrap resampling across subject pair RSMs to estimate the sampling distribution. Different visual stimuli evoked distinct BOLD patterns in both regions from 2 months of age, assessed by correlation to an RSM that contrasts within-exemplar versus between-exemplar similarities (Extended Data Fig. 2). In EVC, representations were similar in their correlation to this within versus between stimulus model at 2 months of age (Spearman correlation of brain RSM and model RSM *ρ* = 0.321, 95% bootstrap CI: 0.314−0.328), at 9 months of age (*ρ* = 0.333, 95% CI: 0.327−0.339) and in adulthood (*ρ* = 0.322, 95% CI: 0.315−0.330). In VVC, the within versus between image distinction was present at 2 months of age (*ρ* = 0.169, 95% CI: 0.128−0.206), becoming stronger in its representation by 9 months of age (*ρ* = 0.229, 95% CI: 0.201−0.255) and again into adulthood (*ρ* = 0.271, 95% CI: 0.251−0.288). This pattern was preserved when using importance reweighting during bootstrapping to match motion distributions (Extended Data Fig. 3a,b). Our dataset revealed a clear stimulus-specific representational geometry in the infant brain, which shows considerable continuity with adults, while continuing to develop throughout the first year of life. This maturity of individual object representations was evident in EVC and VVC at an age when overt visual categorization is restricted or undetectable^37^, visual acuity is still developing^38^ and experience with the world is limited^39^.

These results show that distinct representations are present for individual images, but it is unclear if this is due to their perceptual similarity or to high-level properties that facilitate category grouping. To investigate this, we quantified the features comprising this relational geometry through comparison with perceptual and categorical model RSMs (Extended Data Fig. 2). Informed by existing behavioral and EEG evidence^21,37^, we hypothesized that there would be a transition from low-level perceptual organization in the infants to high-level categorical organization in adults. According to the bottom-up theory of visual development, this perceptual to categorical transition should occur along the object processing hierarchy of the visual stream. The perceptual RSMs chosen were size, elongation, color and compactness—features that have previously been used to model the trajectory of infant looking time behavior from 4 months to 19 months of age^37^. Semantic models were defined by category membership. These were the within versus between image RSM described above, generalization across different exemplars within the same basic-level category (category RSM), similarity within the global categories of animate versus inanimate objects (animacy RSM) and small versus large inanimate objects. This tripartite animacy distinction is a known organizing principle of adult visual cortex to which infants are not behaviorally sensitive until 10 months of age^37,40^.

**Fig. 3:**
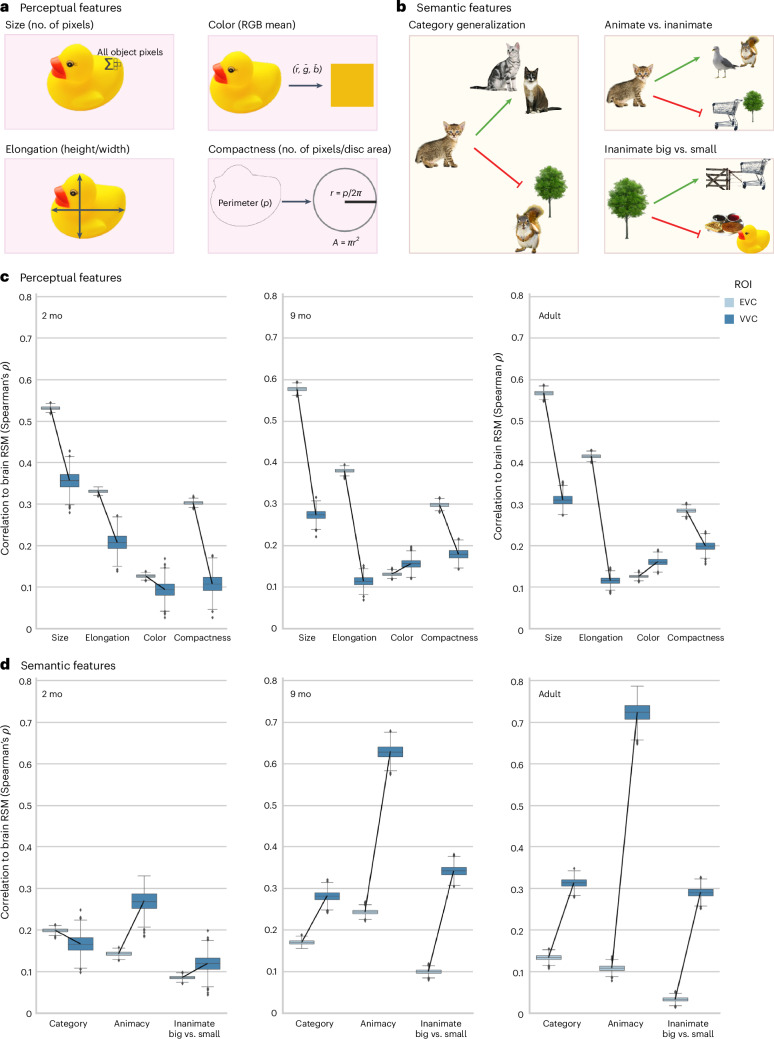
The development of perceptual and semantic feature representations. Perceptual (**a**) and categorical (**b**) features were calculated for each stimulus to construct model RSMs. Created in BioRender: O’doherty, C. (2025): https://BioRender.com/dka6man. Correspondence of visual representations from EVC and VVC to perceptual RSMs (**c**) and semantic feature RSMs (**d**), controlling for the four perceptual features with a partial correlation. Correlations were normalized by the noise ceiling. Boxes are quartiles of the bootstrapped 95% CI, across subject/run pairs (2 months (*n* = 101): 14,108 unique pairs, 9 months (*n* = 44): 1,995 unique pairs; adult (*n* = 17): 136 unique pairs). The lower and upper bounds are the 25th and 75th percentiles; the middle line is the median; and whiskers extend to 1.5 × IQR. Individual points are outliers. mo, months old. Source data

All features were present in each age group (Fig. 3c,d), including distinct categorical representations across visual cortex at 2 months of age, which strengthened throughout the first year of life. Correspondence to the semantic feature RSMs controlled for the four perceptual features through a partial correlation. Perceptual and categorical representations were present in the ventral stream for all the age groups; rather than a transition from the representation being driven by low-level visual features to high-level information, visual cortex begins with features spanning a range of complexities, which is fine-tuned with age. These findings persisted when replicating the tests in a smaller sample with restricted motion distributions (Extended Data Fig. 3a,b). However, we found no effect of longitudinal similarity when comparing the same infants at 2 months and 9 months of age (Extended Data Fig. 3c).

**Fig. 4:**
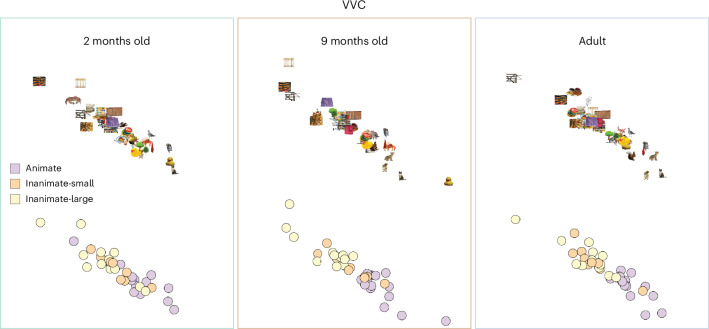
Global organization by animacy emerges in the ventral stream throughout the first year of life. MDS plots of the VVC representations in Fig. 2. The embedding space of the pairwise distances between all images is shown in each age group as well as their global category membership. The rubber duck category was grouped with animate objects in all age groups’ visual representations and has been defined as such here. To ensure similar projections in each plot, embedding models were fit across the ROIs VVC and V1, then separately refined for VVC collapsed across ages and, finally, for age.

Infants represented categories from 2 months of age, but were they encoding the organizing principles that are present in the adult VVS, such as animacy and real-world size?^40^ We found evidence for this much earlier in the brain than has been previously reported from looking time. The animacy distinction was present in the VVC from 2 months of age to a similar degree as category representations (Fig. 3d). However, this was refined with age and experience (Fig. 4) (partial Spearman correlation, controlling for the four perceptual features in 2-month-old infants, *ρ* = 0.198, 95% CI: 0.164−0.233; in 9-month-old infants, *ρ* = 0.552, 95% CI: 0.522−0.581; and in adults, *ρ* = 0.657, 95% CI: 0.613−0.696, significant change with each age). Representation of real-world size was weaker but followed a similar developmental trajectory (partial correlation to inanimate-large versus inaminate-small model in 2-month-old infants, *ρ* = 0.089, 95% CI: 0.057−0.118; in 9-month-old infants, *ρ* = 0.299, 95% CI: 0.278−0.322; and in adults, *ρ* = 0.263, 95% CI: 0.242−0.282). We found that basic-level categories are present in the ventral stream by 2 months of age, with an initial template of organization by animacy and real-world size. This global organization is further distinguished by the latter half of the first year, as it becomes evident in looking behavior^34^.

## LO regions are later to mature

**Fig. 5:**
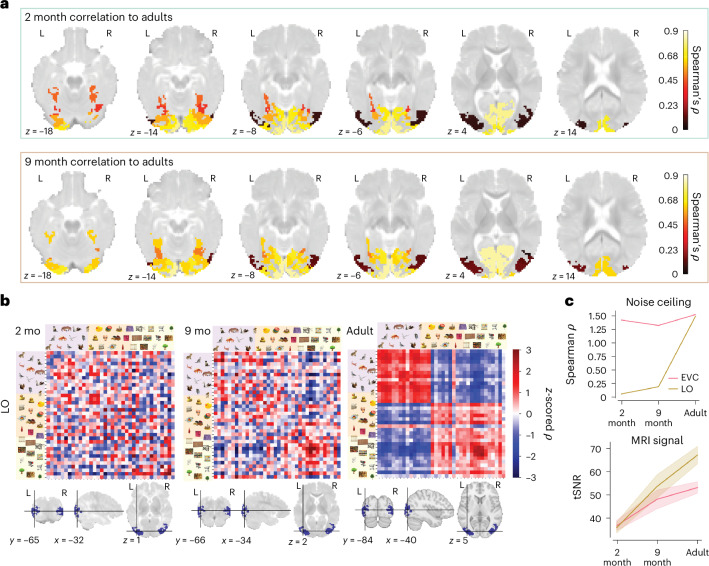
Delayed maturation of lateral object-selective cortex. **a**, Correlations in each visual region of infant and adult representations. To compare equivalent slices at 2 months and 9 months of age, the 2-month-old atlas was transformed into 9-month space. **b**, Group average visual representations for LO. *z*-scored responses are ordered as in Fig. 2. Raw correlation ranges in 2-month-old infants (−0.0053 to 0.0089), 9-month-old infants (−0.0130 to 0.0176) and adults (−0.0712 to 0.0925). **c**, The split-half reliability in LO was extremely low in the infant cohorts relative to a mature medial region, such as EVC. This was dissociated from the MRI tSNR, which increased with age at similar rates in EVC and LO, revealing that the lack of structure seen in **b** was due to inconsistent visual representations across infant participants and not poor signal in these regions. Solid lines in the top panel are the Fisher-transformed split-half reliability; solid lines in the bottom panel are the mean tSNR; and error bands are the 95% CI across runs (169 runs in 2-month-old infants, 64 runs in 9-month-old infants and 51 runs in adults). Source data

The above analyses used an aggregated ROI approach to distinguish trends in two large divisions of the ventral stream, comprising many functionally distinct regions. We expanded this analysis to characterize the maturity of visual representations across finer parcellations of visual cortex. To assess the maturity of representations within each region, the group average infant RSMs were correlated to group average RSMs in the corresponding ROI, bootstrapping across infants to obtain CIs (Fig. 5a). Maturity was highest in early visual regions (correlation to adults in 2-month-old V1, *ρ* = 0.788, 95% CI: 0.782−0.794; 9-month-old V1, *ρ* = 0.809, 95% CI: 0.800−0.819) relative to anterior ventral visual regions such as the parahippocampal cortex (PHC) (correlation to adults in 2-month-old infants, *ρ* = 0.424, 95% CI: 0.390−0.457), with continued maturation throughout the first year of life (9-month-old infant PHC, *ρ* = 0.575, 95% CI: 0.546−0.601). However, maturity differed most noticeably along the medial to lateral axis, in volumetric space, with LO regions being particularly immature (correlation to adults in 2-month-old infants, *ρ* = 0.120, 95% CI: 0.076−0.160; in 9-month-old infants, *ρ* = 0.298, 95% CI: 0.250−0.344).

**Extended Data Fig. 4:**
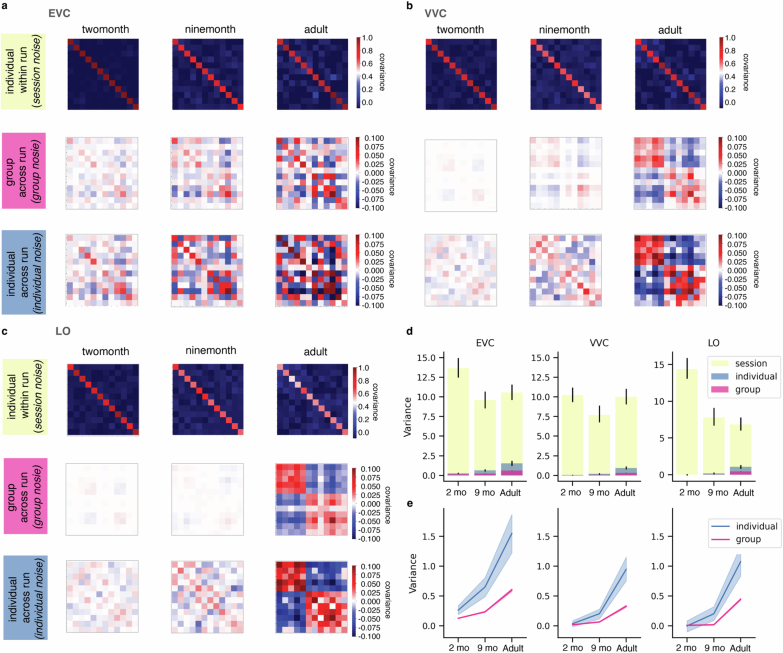
Variance partitioning analysis for group, individual and session-based variance. **(a,b,c)** Group average pairwise covariances across 12 categories’ beta estimates in EVC, VVC and LO respectively using a 12-category GLM that is collapsed across exemplars. Pairwise comparisons were calculated within subjects/within run (331 unique pairs in n = 101 2-month-olds, 128 pairs in n = 44 9-month-olds, 51 pairs in n = 17 adults), across subjects/runs (54125 unique pairs in 2-month-olds, 7980 pairs in 9-month-olds, 1224 pairs in adults) and within subject/across runs (490 unique pairs in 2-month-olds, 148 pairs in 9-month-olds, 51 pairs in adults) to partition the session, group and subject variances respectively. Variance estimates are not z-scored, and X and Y axes of the matrices are identical, ordered by stimulus category. **(d)** Overall estimate of within run (session), across subjects (group) and across run (individual) noise calculated using the trace of the RSMs in (a) and (b) for EVC, VVC and LO. All sources of noise varied with age. Error bars are the 95% confidence intervals, calculated by bootstrap resampling across subject/run pairs in the group average RSM. **(e)** The same noise estimates in (c) and (d), with only the group and individual level variance plot to illustrate developmental changes in shared and idiosyncratic noise. The solid line is the true value of the trace in the covariance similarity matrices; the error bands are shaded between the 95% CI values at each categorical age variable on the x axis. Source data

In stark contrast to adults, RSMs from LO showed no evidence of category or animacy organization in the infant cohorts (Fig. 5b), despite this region being selective for intact versus scrambled objects in adults^41,42^. Visual representations were not reliable in infant LO, as indicated by the split-half noise ceiling (2-month-old infants: *ρ*(51) = 0.032; 9-month-old infants: *ρ*(22) = 0.301; adults: *ρ*(9) = 0.957). We hypothesized that this was due to some source of region-specific MRI measurement noise. Alternatively, if infant LO was not sensitive to the differences between our stimuli, this would also lead to unreliable signal in the RSM. To distinguish these potential causes, we assessed measurement noise using the temporal signal-to-noise ratio (tSNR; Extended Data Table 2) of the regional BOLD timecourses. These were similar between LO and EVC, suggesting similar data quality (Fig. 5c). Furthermore, a variance partitioning analysis showed similar developmental trajectories for EVC, VVC and LO (Extended Data Fig. 4). We, therefore, interpret the results in infant LO as a true lack of response to visual differences. The developmental transition of feature complexity in LO was not necessary for the category representations to be present in anterior VVC, contrary to a bottom-up model of visual development.

## DNNs model infant visual cortex

**Fig. 6:**
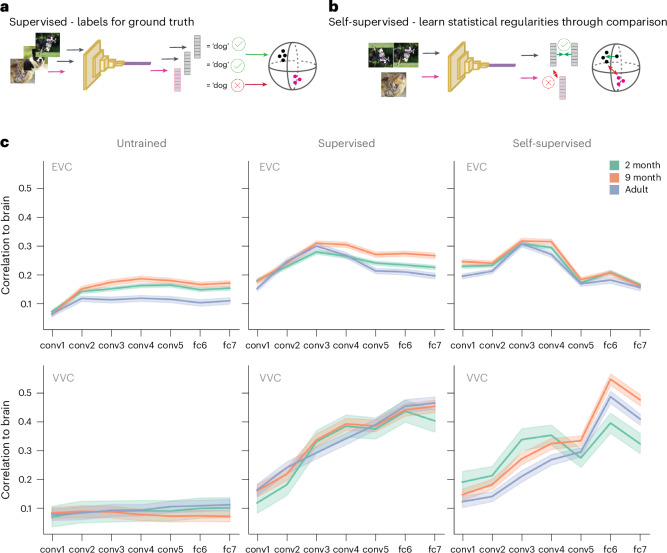
DNNs model visual representations from infancy to adulthood. **a**,**b**, Activations were calculated from DNNs trained with two different algorithms: supervised, trained on a fully labeled object recognition task, and self-supervised, trained using instance protocol contrastive learning. **c**, Layerwise Spearman correlations between the DNN AlexNet and visual cortex. Correlations were normalized by the noise ceiling within each age group and ROI. Solid lines are the mean, and error bands depict the 95% CI, calculated using bootstrap resampling across subject/run pairs (2 months (*n* = 101): 14,108 unique pairs; 9 months (*n* = 44): 1,995 unique pairs; adult (*n* = 17): 136 unique pairs). conv, convolutional; fc, fully connected. Source data

**Extended Data Fig. 5:**
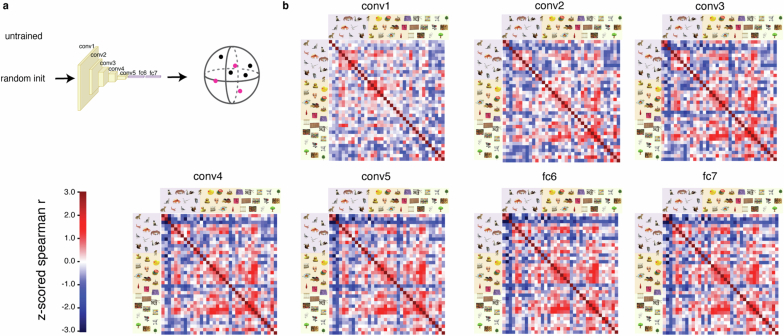
RSMs for untrained AlexNet. **(a)** AlexNet was initialised with random weights and had no exposure to visual stimuli. Layer wise activations were calculated in response to the 36 stimuli, while keeping weights frozen. **(b)** Untrained RSMs for each layer. X and Y axes are the 36 objects, nested by tripartite class (3), category (4) and exemplar (3). Pairwise correlations were calculated between activations in response to each stimulus. We plot the z-scored correlation to highlight representational content rather than strength, and to aid comparison to brain-derived RSMs. Raw correlation ranges: all diagonal values are 1, indicating within exemplar comparisons, off-diagonal ranges were conv1: (−0.1,0.5), conv2: (0.1,0.7), conv3: (0.3,0.9), conv4: (0.4,0.9), conv5: (0.4,0.9), fc6: (0.6,1) fc7: (0.7,1). conv, convolutional. fc, fully connected. Source data

**Extended Data Fig. 6:**
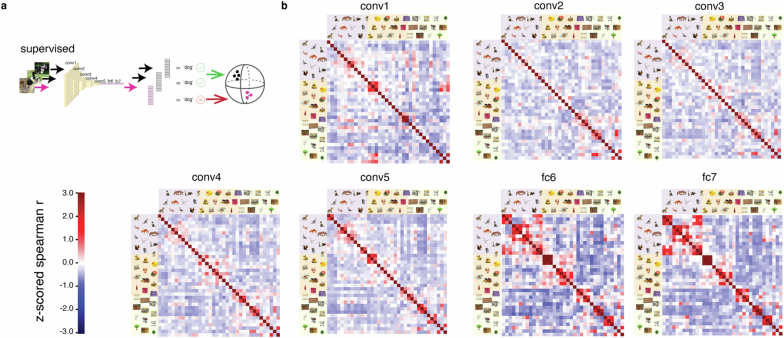
RSMs for supervised AlexNet. **(a)** AlexNet was initialised with the pre-trained weights, learned during a supervised ImageNet recognition task. The model had access to all image labels during training. **(b)** Supervised RSMs for each layer, calculated using correlation between the activation vectors of the model in response to each stimulus image. X and Y axes are the 36 objects, nested by tripartite class, category and exemplar. Pairwise correlations were calculated between activations in response to each stimulus. We plot the z-scored correlation to highlight representational content rather than strength, and to aid comparison to brain-derived RSMs. Raw correlation ranges: all diagonal values are 1, off-diagonal values: conv1: (0,0.5), conv2: (0,0.3), conv3: (0,0.3), conv4: (0,0.3), conv5: (0,0.4), fc6: (−0.1, 0.7), fc7: (−0.1,0.8). conv, convolutional. fc, fully connected. Source data

**Extended Data Fig. 7:**
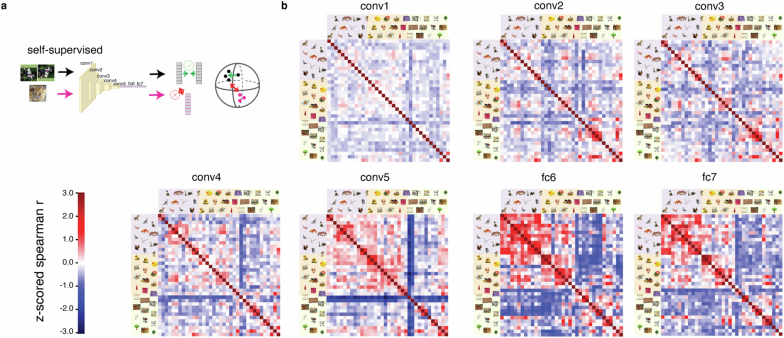
RSMs for self-supervised AlexNet. **(a)** The self-supervised model was trained with Instance Protocol Contrastive Learning (https://github.com/harvard-visionlab/open_ipcl). The algorithm learned only from natural image structure by bringing together augmented versions of an image closer to an average “prototype” of the image in embedding space, while keeping the image’s representation distinct from recently encountered images in memory. Activation vectors were calculated in response to each of the 36 images, while keeping training weights frozen. **(b)** Unsupervised RSMs for each layer, calculated using pairwise correlations between each image’s activation vector. X and Y axes are the 36 objects, nested by tripartite class, category and exemplar. We plot the z-scored correlation to highlight representational content rather than strength, and to aid comparison to brain-derived RSMs. Raw correlation ranges: all diagonal values are 1, off-diagonal ranges: conv1: (0,0.3), conv2: (0,0.5), conv3: (0.1,0.5), conv4: (0.2,0.6), conv5: (0.8,0.9), fc6: (−0.1,0.8), fc7: (0,0.7). conv, convolutional. fc, fully connected. Source data

We found the presence of high-level features in infant visual representations, as defined by within versus between category distinctions. However, category representations could be shaped by many visual features with potentially complex multidimensional tuning in feature space. DNNs have shown great success in capturing these complex feature manifolds from the statistics of visual input, learning representations of objects that are simultaneously successful for recognition and relevant for adult visual cortex^27^. To date, parallels to these models have only been drawn to the ‘fully trained’ adult brain. With our dataset characterizing infant visual representations, we can now compare the ‘learning’ human brain to the ‘learning’ model. Here, we tested if deep-learning-based models of visual recognition can capture infant neural responses (Fig. 6a). Using the 36 images from the fMRI experiment, we calculated activations from a set of models with an AlexNet architecture (Extended Data Figs. 5–7) by testing the extremes of the training process: untrained and fully trained. The architecture of untrained neural networks can provide sufficient inductive biases for capturing object-relevant information^43^, but training the network weights through visual input to the model confers an important advantage for representation learning. By testing correspondences to the models at these two stages, we can delineate the contribution of features that are learnable from the statistics of visual experience for explaining brain representations.

**Extended Data Fig. 8:**
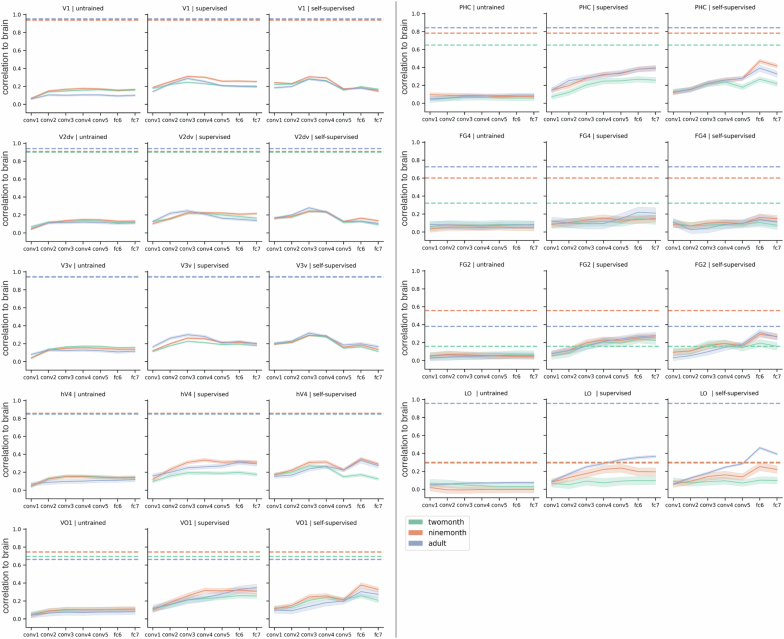
DNN Correlations to individual ROIs. Layer-wise Spearman correlations between the DNN AlexNet and visual cortex. Horizontal dash lines indicate the noise ceiling within each age group and ROI, rather than normalisation, as division by a low reliability score in some individual regions is not valid. This would artificially inflate the resulting correlation when visual responses are not consistent across the group for this region. The sampling distribution of Spearman correlations was estimated using bootstrap resampling across individual subject/run pair RSMs (2-months (n = 101): 14108 unique subject/run pairs, 9-months (n = 44): 1995 pairs, adults (n = 17): 136 pairs). Solid lines are the mean bootstrap correlation value and error bands depict the 95% confidence interval. conv, convolutional. fc, fully connected. Source data

Although infant representations in EVC correlated more with an untrained DNN than adult representations at both 2 months and 9 months of age (Fig. 6c), a fully trained network outperformed the untrained model across all age groups. This demonstrates that, from as early as 2 months of age, features learned from the statistics of visual input—used by machines for object classification—are important for explaining visual representations. Even with less visual experience^44^, infants’ representations are sophisticated enough to correspond well with those from a fully trained neural network. This DNN model underwent supervised training, where each image had a corresponding label, something pre-verbal infants would not have had access to. Instead, infants are sensitive to comparisons and patterns within the stream of sensory input^45,46^, aligning better with self-supervised training. To test if the observed effect generalized to DNNs trained without labels, we compared to a DNN trained with a self-supervised algorithm^47^. Infants showed similar patterns of correlation to the model regardless of learning algorithm, with the expected hierarchical correspondence between brain and model emerging along the VVS^26^ (Extended Data Fig. 8). Early visual cortex peaked in its correspondence to the DNN in earlier layers. In all age groups, VVC representations were most similar to deep layers that capture higher-level visual properties of the images, facilitating object classification. Notably, some layers of the model trained with self-supervised learning explained infants’ VVC significantly better than adults’ when compared to the supervised model. This demonstrates that different DNNs are better models for different developmental stages, opening the future possibility to identify the learning model that best explains infant brain data.

## No evidence for bottom-up development of the VVS

**Fig. 7:**
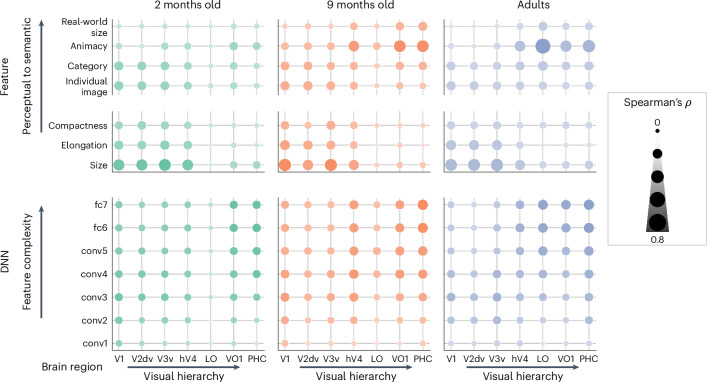
Developmental cascades of feature complexity along the ventral stream. Scatter plots showing correlations to the features used in RSA and each layer of supervised AlexNet versus regions along the visual hierarchy. The size of each dot depicts the Spearman correlation between model and brain RSMs, standardized across all ages and test types. Correlations were not normalized by the noise ceiling due to invalidity in individual regions where there is lack of reliable signal, such as LO. The opacity of each dot ranges from the minimum to maximum value within a subplot. VVS regions become more functionally specialized through development, but high-level visual features captured by categorical features and deep layers of a neural network are present throughout the visual hierarchy from 2 months of age. conv, convolutional; fc, fully connected. Source data

Our results have shown that high-level visual features are present across the ventral stream at 2 months of age, with refinement of the functional distinctions between low-level and high-level regions throughout development. ln Fig. 7, we summarize our findings and further highlight this functional specialization along the visual hierarchy. Early visual regions in the 2-month-old brain represent features that span a range of complexities, captured by perceptual and categorical image features and many neural network layers. In the older age groups, EVC representations become specialized toward low-level visual features. In VVC, many features are represented at 2 months of age, with a bias toward categorical features. These high-level responses become functionally distinct in VVC with age and experience, especially for global organization by animacy. The influence of complex features captured by DNN layers on early visual representations becomes less pronounced with age. LO, a mid-level processing region, shows a protracted development relative to other object-selective regions. Thus, we found no evidence for a bottom-up transition from simple to complex features along the visual processing hierarchy. Instead, high-level feature representations are present in the ventral stream from 2 months of age and are fine-tuned with age and experience. When considering the maturation from medial toward lateral visual regions, we found a protracted development of object-selective regions in LO, which lies posterior to the VVC, indicating non-hierarchical development of visual regions involved in object perception.

## Discussion

Using a broad set of stimuli, we found that infant VVC contained category and animacy information at 2 months of age, which was not explained by low-level visual features. This category structure appears in ventral regions of the visual hierarchy before emerging in lateral regions, revealing that category representations in visual cortex do not develop in a bottom-up manner. In agreement with retinotopic studies^48^, early medial regions are the most mature at 2 months of age. The features encoded in visual representations are refined with age and experience rather than a developmental transition from simple to complex feature encoding.

Converging evidence now suggests that object processing in LO is later to mature. Previous infant fMRI studies using face and place stimuli found that focal regions of selectivity are present in ventral regions on the fusiform gyrus, such as fusiform face area (FFA) and parahippocampal place area (PPA), with no evidence for selectivity in lateral precursor regions occipital face area (OFA) and occipital place area (OPA)^33,49^. Shape processing, but not object selectivity, has been demonstrated in LO of 6-month-old infants using functional near-infrared spectroscopy (fNIRS)^50^, and steady-state visual evoked responses to scrambled versus intact objects are immature at this age^51^. Moreover, the improved spatial resolution of fMRI highlights that the developmental transition in feature complexity previously reported from behavioral and MEG/EEG studies^20,21^ may be driven by lateral visual regions rather than immature function of category processing along the ventral stream. LO is an important object-selective region, so why might it show a protracted development? Neural activity in this region is modulated by attentional shifts^52^, facilitates scene recognition from component objects^53^ and has closely overlapping representations for object classes and their intended motor function^54^. The contributions of these cross-modal and higher-order cognitive processes might require more experience; improved motor abilities may be necessary for an object’s intended action to be represented^55^; or sufficient activation of infants’ attention networks^56^ may constrain the complete emergence of object recognition. Indeed, the long-range connectivity of future category regions on the ventral surface has been found from early infancy^57,58^, whereas myelination of white matter tracts occurs later in lateral regions^59^.

Infant ventral visual representations corresponded with a fully trained DNN, indicating that complex features sufficient for classification in machines are present as early as 2 months of age. A critical question for the future will be to establish when this representation is set up. Is it rapidly learned in 2 months of visual experience, or is there a biological mechanism that establishes VVC with the ability to distinguish categories from birth? An additional 7 months of visual experience is likely contributing to the fine-tuning of VVC seen between 2 months and 9 months. However, the notable presence of distinct category responses already at 2 months—despite the paucity of visual acuity and color vision during this period^38,60^—calls into question if the emergence of this function is entirely experience dependent. There are known examples of neural adaptations to the statistics of the visual world. For example, the center-surround structure of retinal ganglion cells provides efficient coding for spatially extended patches in the visual environment by performing edge detection^61^. We propose that neural adaptation has been extended to the identification of visual categories and their invariances. A mechanistic interplay between innate responsiveness to categories, and sufficient flexibility for learning, could facilitate rapid adaptation to one’s own sensory world. Infants show preference toward culturally relevant objects and backgrounds by 12 months of age^62^, and face-selective regions in the ventral cortex of congenitally blind individuals are known to be highly plastic^8,63^. Perhaps a developmental dissociation between distributed representations and category-selective focal regions, as has also been described in children^64^, facilitates this interplay between intrinsic and extrinsic factors. If the distributed manifold of VVC representations begins with the necessary structure to distinguish categories in general, approximately identifying what is seen, then learning the detailed visual features necessary for understanding idiosyncratic experience could be made much easier.

Our dataset allowed DNN models of ventral cortex to be applied to infants. Even more than for adult vision research, DNNs offer the potential for novel insight into visual development. The parallel between infant learning and machine learning is of increasing interest for researchers in both fields^65,66^, with recent work showing that video data from an infant’s perspective provide the necessary structure to learn word−visual referents^67^. Our work brings this approach a step earlier in the developmental process, using DNNs to characterize the visual representations to which words are later attached. By incorporating rich awake infant neuroimaging paradigms into our toolkit, we unlock the substantial potential of computational modeling to understand the learning mechanisms implemented in the developing brain, allowing us to gain further insight of the first year of life as a time of intricate development and complex brain function.

## Methods

### Participants

Infants were recruited from participating maternity hospitals and attended two scanning sessions at Trinity College Institute of Neuroscience. Caregivers who expressed interest in the study when approached on the postpartum ward were contacted 1 month after the infantsʼ due date, at which time they decided if they wanted to participate. The study details and participant information leaflet were sent to caregivers to review and were explained clearly on the day of scanning. The first scan was scheduled for as close as possible to when the infant was 2 months corrected gestational age (CGA) and another at 9 months CGA. At this session, caregivers provided informed consent on behalf of their infant. Participants’ travel expenses consequent to the participation in the study were paid for in cash, and they were provided after every session with a One4All voucher for the amount of €35 to be spent at their will. Twenty-eight participants’ caregivers chose not to continue in the study at the 9-month follow-up, and three infants were not invited back due to poor tolerance of the scanning at 2 months. Awake fMRI was successful in 97% of 2-month-old infants and in 64% of 9-month-old infants. The final dataset consisted of 130 2-month-old infants (52 female, 78 male, 1.5–4.7 months CGA, mean = 2.4 months) and 66 9-month-old infants (31 female, 35 male, 7.5–10.9 months CGA, mean = 9.3 months) and was composed of infants born healthy at full term (*n* = 101 2 months, *n* = 55 9 months) as well as a smaller proportion who were born pre-term and spent time in the neonatal intensive care unit (NICU) (*n* = 29 2 months, *n* = 11 9 months). For this initial study, both groups were combined, but results were validated in a subset of only full-term infants. Future work will test for differences between infants born full-term and pre-term. Ethical approval was obtained from the Trinity College Dublin School of Psychology Research Ethics Committee, the Rotunda Ethics Committee and the Coombe Ethics Committee.

### Infant fMRI data collection

We iterated upon scanning procedures from pioneering methods^23^, using projection onto the bore ceiling and the bottom half of the head coil to allow the infant an uninterrupted view. Additionally, for participants at the 2-month timepoint, a four-channel surface coil was placed above the infant’s forehead. Similarly to Ellis et al.^23^, we built our own stimulus display program using PsychoPy^68^ version 2022.1.4 in Python version 3.7.12, which was essential to successful scanning, enabling constant stimulation as well as fast and flexible switching between tasks depending on the temperament of the infant. We deployed an in-bore MRI-compatible camera (MRC Systems HighResolution), which streamed a video of the infant’s face to the scanning team and the parents, allowing us to monitor the infant’s comfort levels closely, put caregivers at ease and record gaze.

MRI data were collected on a Siemens MAGNETOM Prisma 3T scanner using the posterior array of the 64-channel head/neck adult coil and four-channel flex coil in the 2-month-old infants. The task-based fMRI sequences used a multi-band accelerator factor of 4, voxel size 3 ×3 × 3 mm, field of view (FOV) 192 × 192 mm, phase encoding direction anterior to posterior, repetition time (TR) 610 ms, echo time (TE) 32 ms, flip angle 40°, echo spacing 0.54 ms and 36 slices per volume. In total, 510 volumes were collected for the pictures task. Two short reference sequences of single-band spin-echo volumes with opposite (anterior/posterior and posterior/anterior) phase encoding directions were also collected, with TR 2,260 ms, TE 32 ms and identical geometry to the fMRI, with a scanning time of 29 seconds. These two reference scans were used to apply susceptibility distortion correction using TOPUP from the Functional Magnetic Resonance Imaging of the Brain (FMRIB) Software Library (FSL). T2-weighted images were a noise-reduced scan consisting of the following parameters: 100 contiguous near-axial slices, GRAPPA acceleration factor of 3, voxel size 1 mm × 1 mm × 1 mm, coverage of the whole head and FOV 192 mm, TR 6,090 ms, TE 84 ms, flip angle 150°, echo spacing 12 ms and duration 4 minutes, 5 seconds. Later in the study, a T1 MPRAGE sequence was added with 144 sagittal slices, voxel size 1 mm × 1 mm × 1 mm, coverage of the whole head and FOV 256 mm, TR 2,300 ms, TE 2.98 ms, TI 900 ms, flip angle 9°, echo spacing 7.1 ms and duration 6 minutes, 28 seconds. The set of data collected in each session varied depending on infant temperament, technical capacity and time constraints. The results and data of other tasks will be released in future work.

### Preprocessing

Extensive manual data curation and quality control were crucial in standardizing the infant fMRI data. MRI data were preprocessed using an in-house pipeline written in Python 3.8.10 with the NiPype 1.8.5 processing framework^69^ to avail of the neuroimaging software packages FSL 6.0 (ref. ^70^) and ANTs 2.4.4 (ref. ^71^). Two fieldmap reference scans with opposite phase encoding (anterior/posterior and posterior/anterior) were input to FSL TOPUP^72^ to estimate the susceptibility-induced off-resonance field. Echo planar imaging (EPI) images were motion corrected to a middle reference volume using FSL MCFLIRT^73^ and converted to SPM format from which FWD and DVARS were calculated. The field distortion estimation from TOPUP was then applied to the EPI data to correct for susceptibility distortions. Brain images were co-registered to the mean of a chosen reference scan using an affine transform with FSL FLIRT^74^. Due to limited movement in the infants when they fell asleep, resting-state scans were the preferred choice for the functional reference. In the absence of a resting-state scan, awake fMRI runs were used instead. The degrees of freedom (DOF) used for the affine transform were chosen by manual inspection for each infant, with the vast majority of participants having a successful registration with DOF = 12. Scans in native space were then normalized to a common space with FSL FLIRT, using an age-appropriate NIHPD template^75^. The cytoarchitectonic Julich atlas^76^ was transformed into the infant space to define various ROIs. Regions in this atlas were previously validated to overlap with functionally defined retinotopic areas^77,78^, enabling us to select a subset of visual ROIs. These were V1/hOc1, V2dv/hOc2, V3v/hOc3v, hV4/hOc4v, VO1/FG1 and PHC/FG3, all referred to in-text by their functional name. Additional cytoarchitecturally defined regions were FG2 and FG4 on the fusiform gyrus and lateral object-selective regions LOC/hOc5, LOCla/hOc4la and LOClp/hOc4lp, which were validated to overlap with object regions using https://neurosynth.org/. Aggregated ROIs were used to provide broadly interpretable measures along the ventral stream. These were EVC (V1 + V2dv + V3v), VVC (VO1 + PHC + FG2 + FG4) and LO (LOC + LOCla + LOClp). ROIs are plotted in adult space in Extended Data Fig. 1d.

### Stimuli selection and adult piloting

Picture stimuli were three exemplars from 12 categories, chosen to be something an infant may encounter in the first year of life, in person or in books, to correspond to words with an early age of acquisition and to be balanced across the animate, small inanimate and large inanimate categories. These were cat, crab, bird, squirrel, rubber duck, dishware, fence, food, supermarket shelves, shopping cart, towel and tree. Given that category-selective responses to faces and scenes have previously been shown in infants^22,33^, and that these categories could be tested in another task, we chose to prioritize a broader range of categories. Piloting in adults helped inform the final 12 categories tested. Adult participants (*n* = 18) were recruited through advertisements and word of mouth at Trinity College Dublin under the approval of the School of Psychology Ethics Committee. One participant was excluded due to technical difficulties during the session, resulting in a final sample of *n* = 17. Scanning consisted of three fMRI runs of the pictures task, with twice as many stimuli as in the infant paradigm and only one repetition of a stimulus per run (6 contexts × 4 object types × 3 instances × 1 repetition). The final infant stimuli were chosen from this larger set of adult pilot stimuli through an iterative modeling process to find the stimuli that gave the strongest and most separable brain responses in adults while adhering to known organizing principles of the ventral stream.

Adult MRI data were collected using the full 64-channel Siemens Head/Neck coil. The acquisition parameters were as follows: a multi-band acceleration factor of 4 was used with a voxel size of 3 × 3 × 3 mm and a FOV of 224 × 245 mm; the phase encoding direction was left to right; repetition time was 656 ms and echo time was 30 ms; flip angle was 50°; and echo spacing was 0.54 ms. Forty slices were collected per volume, and 561 volumes were taken due to the longer design for this piloting study. As per the infant protocol, Digital Imaging and Communications in Medicine (DICOM) data were converted into Brain Imaging Data Structure (BIDS) format^79^ using HeuDiConv version 0.10.0 (ref. ^80^). The adult dataset was preprocessed using fMRIPrep^81^ with FSL.

### Task fMRI

Pictures appeared in pseudorandom order against a black background for 3 seconds followed by a fixation cross, with the jittered stimulus onset asynchrony ranging between 3.5 seconds and 4.5 seconds. Presentation was considered pseudorandom due to a restriction that one exemplar from each category appeared before there was a second exemplar of a given category. The order of the exemplars within each of these category repetitions was always randomized across subjects and runs. Images started at half of their final size and loomed toward participants, increasing logarithmically in time, doubling in size within the presentation window. There were three exemplars for each of the 12 categories, and each was repeated twice per fMRI run. This gave a final design of 12 categories × 3 exemplars × 2 repetitions, totaling 5 minutes of scanning. During the task, a series of short nursery rhymes played through the infant headphones to maintain engagement. Images were obtained online, selecting exemplars from a variety of viewpoints for each category. Backgrounds were removed, and the images were rescaled so that the largest dimension, either width or height, touched the image border. Then, images were resized and padded to a standard size of 640 × 360 pixels and pre-distorted to ensure that they appeared normal when displayed on the scanner’s curved surface, given the projection geometry.

### First-level model and multivariate pattern analysis

A volumetric whole-brain GLM was fit for each functional run with custom Python code using the Nilearn package (version 0.9.2). A design matrix was constructed with 36 regressors of interest, one for each object, convolved with a canonical Glover HRF function as in previous awake infant fMRI studies^23,48,82^. To account for motion, the FWD values calculated during preprocessing were used to add spike nuisance regressors, giving censoring in frames that exceeded a threshold of 1.5 mm, thereby removing these frames from parameter estimation. This method was previously shown to lead to statistical improvements in high-motion cohorts^83^. If more than 50% of the frames in a run were above this motion threshold, the entire run was discarded from further analyses. Three translation and three rotation motion regressors calculated during preprocessing were included as covariates as well as a linear trend regressor and a cosine drift model with a high-pass filter of 0.01 Hz. This rigorous motion correction resulted in a strict final sample of *n* = 101 2-month-old infants (64 male, 37 female; median CGA = 2.33 months; interquartile range (IQR): 2.17−2.67 months) and *n* = 44 9-month-old infants (22 male, 22 female; median CGA = 9.10 months; IQR: 8.92−9.5 months) included in multivariate pattern analysis (MVPA). The youngest/oldest infants in each group were outliers at 1.53/4.73 months and 7.46/10.77 months, respectively, but CGA was not found to drive results within each group. We, therefore, chose to group infants according to their scanning timepoint of 2 months or 9 months.

The runwise parameter estimates (*β* values) for each condition within each ROI were calculated as well as the variance/covariance (vcov) matrix across timepoints of the design, multiplied by the residual mean square image to estimate dispersion. To control for differences in baseline signal within each fMRI run, we performed run-level mean centering of estimates by subtracting each voxel’s run-level mean across all trials from all estimates within a run, as was shown to improve MVPA results^84^. Due to the high motion in some infant runs and the use of censoring, a small percentage of model fits resulted in unstable parameter estimates and very noisy *β* values, which were corrected using the vcov estimates. If a particular voxel had a vcov dispersion greater than 10, chosen by cross-validated effect on the *β* distribution, it was excluded from the MVPA. The proportion of voxels excluded from the aggregate ROIs in each age group were as follows. Average proportion excluded in 2-month-old infants: EVC median = 3.79% (IQR: 0.76– 9.56%); VVC median = 3.47% (IQR: 0.77−8.43%); and LO median = 1.7% (IQR: 0.24−8.76%). In 9-month-old infants, this was EVC median = 1.64% (IQR: 0.72−4.88%); VVC median = 2.25% (IQR: 0.26−8.18%); and LO median = 0.39% (IQR: 0.00−2.06%). In adults, this was EVC median = 7.29% (IQR: 5.12−8.66%); VVC median = 8.98% (IQR: 4.64−13.73%); and LO median = 2.66% (IQR: 1.35−4.05%).

The pairwise correlations between the 36 objects’ voxelwise response patterns were calculated across all unique pairs of subjects and runs (2 months: 14,108 unique subject/run pairs; 9 months: 1,995 unique subject/run pairs; adult: 136 unique subject/run pairs). These across-subject/pair RSMs were then averaged to calculate a group visual representation, ensuring that observed patterns were restricted to signals that were common across the group. Distances were centered about 0, which resulted in highly consistent group average correlations but small magnitudes that varied in scale with age (2 months (−0.027 to 0.030); 9 months (−0.053 to 0.073); adult (−0.11 to 0.14)). Therefore, we *z*-scored when plotting the RSMs to focus on representational content in terms of relational geometry rather than strength. Distances in representational space were visualized by projecting into a two-dimensional space using multidimensional scaling (MDS). To ensure that these visualizations were comparable across age groups, they were aligned hierarchically while minimizing stress using the SMACOF algorithm. MDS embedding models were first fit across ages and V1/VVC, then separately refined for each region (collapsed across ages) and, finally, for age.

To obtain test statistics in all analyses, RSMs were randomly sampled across subject/run pairs, with replacement, prior to averaging. In this way, the sampling distribution was estimated using bootstrap resampling (1,000 bootstraps) across pairs of subjects, enabling generalization to an unseen set of subject/run pairs. When comparing across age groups, bootstrap resampling with importance reweighting was implemented to ensure that results were not driven by differences in their extent of motion or NICU membership. Each individual from a ‘source’ age group was reweighted during sampling so that the resulting distribution of FWD matched a ‘target’ age group. NICU graduates were excluded. To do this, within each age group, participants’ runs were binned according to FWD (mean FWD in 2-month-old infants: 0.912 mm; in 9-month-old infants: 0.532 mm). Motion was lowest in the adult distribution (0.177 mm), which was a subset of the infant distributions, so this was used as a target. Individuals within each FWD bin were resampled with a probability proportional to the ratio of the number of individuals in that bin in the target distribution, divided by the source distribution. After resampling, the 2-month RSMs included 61 runs from *n* = 48 infants with mean FWD = 0.314, and the 9-month RSMs included 42 runs from *n* = 30 participants with mean FWD = 0.303 mm. Key findings were all supported within this restricted analysis; results are reported in Extended Data Fig. 3a,b.

Maturity of individual visual regions was measured by correlating the infant group RSMs to the adult group RSM in the corresponding ROI, resampling across infants only. To measure the bound on performance for the computational models from the fMRI data, the split-half noise ceiling was calculated by correlating two group RSMs from split-halves of the dataset within each ROI and age group and adjusting for test length using the Spearman−Brown prophecy formula^85–87^.

### Variance partitioning

Age-related differences in raw correlation values could reflect a strengthening of representational resources but may also be attributed to noisier data from the infant cohort. The across-subject/run pair approach taken ensures that the resulting RSMs are mostly driven by shared signal, but subject-specific and run-specific noise may be contributing to differences across the groups; this would not be detectable here. A variance partitioning approach was taken using RSMs with covariance as the distance metric, instead of correlation, to leverage its additive property. A GLM at the 12-category level was used. This had six repetitions per category in a run, as within-run repetitions of a single stimulus were required, and models with three exemplars and two repetitions were unstable. The trace of RSMs across three types of comparisons gave an estimate of noise: (1) across pairs of subjects (group variance), (2) within subjects but across stimulus repetitions (individual variance) and (3) within subjects but within stimulus repetitions (session variance). Error estimates on the variance partitions were calculated with bootstrap resampling across subjects/runs. Although session-based noise did decrease with age, it was accompanied by an increase in both the individual and group components (Extended Data Fig. 4) It is possible that the increasing idiosyncratic representational alignment could reflect individual experience^88,89^, and increased group variance could reflect common visual experience in the world. However, the origins of the signal strength differences, whether neural or hemodynamic^90^, remained unclear. We, therefore, focused on representational content by *z*-scoring across each RSM.

### RSA

The correlation between visual representations and a set of theoretical feature RSMs was used to assess the presence of perceptual features and hypothesis-based semantic distinctions. The perceptual feature RSMs—to which infants in the 4−19-month range are sensitive^37^—were calculated from 1 minus the pairwise distances between images’ size (number of foreground pixels − number of background pixels), elongation (maximum of height-to-width or width-to-height ratios), color (the average of the three color channels of the image) and compactness (the ratio between the area of the shape and the area of the disc with the same perimeter). Semantic RSMs were built using categorical distinctions—namely, within versus between image (implemented in practice as an identity matrix) and generalization across exemplars but within category (within-exemplar contrast weights are 0; within-category but across-exemplar weights are 1; and across-category weights are −1). Lastly, broad semantic structure was tested using the tripartite animate, inanimate-large and inanimate-small distinction.

The Spearman correlation was calculated between the vectorized upper triangles of brain and feature RSMs, and the sampling distribution was estimated using the same 1,000 bootstrap resamples across subject/run pairs entering the group average RSM. Group differences were calculated by testing significant difference from 0, using the 95% CIs of the difference between the sampling distributions for pairs of age groups. Partial correlations of the semantic models, accounting for all perceptual models as covariates, determined the extent of semantic information encoded in the visual representations beyond basic perceptual features. Final correlation values were expressed as a proportion of the noise ceiling for a given ROI and age groups where appropriate. Although the low split-half reliability could reflect measurement noise, it may also be a true indication of a lack of consistent signal across the group. When comparing the noise ceiling to changes in tSNR, we found the latter to be the case. Thus, when smaller individual ROIs are plotted with unreliable noise ceiling estimates, we do not normalize by this value. All noise ceilings and tSNR measures are reported in Extended Data Tables 1 and 2. The leading diagonal was included in the feature RSA. When excluding the main diagonal for RSA, the trend in the results reported in Fig. 3 were consistent, with slightly diminished correlation values. Results are reported in full in supplementary tables.

To explore longitudinal effects at the individual level, the within-subject/across-repetition RSMs were used. The similarity between the same infant’s RSM at 2 months versus 9 months was compared to other infants. The sample was reduced to *n* = 38 when restricting to infants who had multiple repetitions of useable data at both sessions. As might be expected given the noise at the level of individual infants, the permutation test revealed that 2-month-old infants were no more correlated to themselves at 9 months than they were to a random infant (Extended Data Fig. 3c). Future work and data releases with our videos task and resting-state, structural and cognitive follow-ups at 18 months and 2 years may be preferable for this kind of analysis.

### DNN modeling

Deep learning analyses were written in Python version 3.8 with PyTorch version 2.0.1 and CUDA 11.7, and all models used the convolutional neural network AlexNet^91^ as the architectural backbone. The untrained model weights were randomly initialized using Glorot initialization^92^. The supervised model was initiated using the default pretrained weights from torchvision version 0.15.2, and, finally, the self-supervised model tested here used the pretrained weights provided by the authors of the self-supervised model instance-prototype contrastive learning^47^ (https://github.com/harvard-visionlab/open_ipcl). Both pretrained models have previously been validated as models for the adult VVS^27,28,47^. The 36 experimental stimuli, projected on a gray background, were input to each model, and the activations from each layer were recorded while keeping model weights frozen. RSMs were then calculated using the pairwise correlation between the images’ activation vectors. Finally, we performed RSA using the upper triangle of the model RSMs and the fMRI RSMs, excluding the diagonal. The degree of similarity between the DNN and brain was measured using Spearman correlation with bootstrapping. Statistics were as described in the above section on RSA, and results are reported in full in the supplementary tables.

### Reporting summary

Further information on research design is available in the Nature Portfolio Reporting Summary linked to this article.

## Online content

Any methods, additional references, Nature Portfolio reporting summaries, source data, extended data, supplementary information, acknowledgements, peer review information; details of author contributions and competing interests; and statements of data and code availability are available at 10.1038/s41593-025-02187-8.

## Supplementary information

Supplementary Information The consent form used for the study

Reporting Summary

Supplementary Table 1 Statistics from all RSA and DNN comparisons in each ROI/age group, including group differences and reproduction in a motion-matched subsample of participants

## Source data

Source Data Fig. 1 .csv files used to plot the distributions in Fig. 1e,f.

Source Data Fig. 2 One .csv file for each RSM plot. Only the group average RSMs are provided for each ROI/age group. The full set of RSMs is available at OpenNeuro (OpenNeuro accession number: ds006883, https://doi.org/10.18112/openneuro.ds006883.v1.0.0) under derivatives/foundcog_rdms/. The full bootstrap distribution of correlations used for the violin plots is provided as a .csv. Note that the MDS plots in Fig. 4 are generated from the same RSMs as are provided for Fig. 2.

Source Data Fig. 3 .csv files with the estimated bootstrap distributions for perceptual and semantic feature RSA, used to generate the box plots.

Source Data Fig. 5 .csv file with the bootstrap distributions of correlation to the adult group for individual ROIs in 2-month-old and 9-month-old infants. One .csv for each LO group average RSM. The full set of RSMs is available at OpenNeuro (OpenNeuro accession number: ds006883, https://doi.org/10.18112/openneuro.ds006883.v1.0.0) under derivatives/foundcog_rdms/. .csv files with the tSNR measures for EVC, VVC and LO for each run and split-half reliability for each ROI/age group.

Source Data Fig. 6 .csv files for all model bootstrap distributions for age/layer/ROI.

Source Data Fig. 7 .csv files with the feature and DNN correlations in individual ROIs for each age group. Spearman correlations reported are the mean of the bootstrapped distribution.

Source Data Extended Data Fig./Table 1 One .csv file for each RSM plot. Only the group average RSMs are provided for each ROI/age group. The full set of RSMs is available at OpenNeuro (OpenNeuro accession number: ds006883, https://doi.org/10.18112/openneuro.ds006883.v1.0.0) under derivatives/foundcog_rdms/.

Source Data Extended Data Fig./Table 2 .csv files calculated using the perceptual features for each of the images. One .csv file per categorical RSM is also included, but these were constructed during the analysis pipeline.

Source Data Extended Data Fig./Table 3 Statistical source data comprising .csv files of the bootstrap distributions for all box plots and longitudinal permutation test.

Source Data Extended Data Fig./Table 4 One .csv file for each RSM plot. Only the group average RSMs are provided for each ROI/age group. The full set of RSMs is available at OpenNeuro (OpenNeuro accession number: ds006883, https://doi.org/10.18112/openneuro.ds006883.v1.0.0) under derivatives/foundcog_rdms/. Statistical source data comprising .csv files of the bootstrap distributions for the trace values reported for the variance partitioning analysis.

Source Data Extended Data Fig./Table 5 One .csv file for each RSM plot from seven layers of untrained/randomly initialized AlexNet.

Source Data Extended Data Fig./Table 6 One .csv file for each RSM plot from seven layers of supervised AlexNet.

Source Data Extended Data Fig./Table 7 One .csv file for each RSM plot from seven layers of self-supervised AlexNet.

Source Data Extended Data Fig./Table 8 Statistical source data comprising .csv files of the bootstrap distributions for all AlexNet model/brain comparisons (3 ages × 9 ROIs × 8 layers × 1,000 bootstraps for each model).

## Figures and Tables

**Extended Data Table 1:**
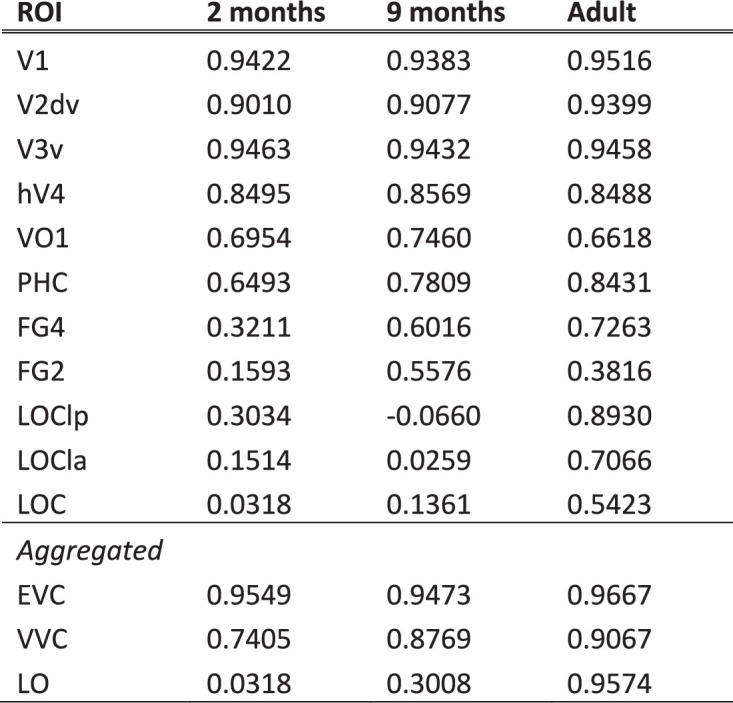
Split-half noise ceilings per ROI/age group

Using all subject/run pair RSMs (2 months (*n* = 101): 14,108 unique subject/run pairs; 9 months (*n* = 44): 1,995 unique subject/run pairs; adults (*n* = 17): 136 unique subject/run pairs), a split-half reliability was calculated for each ROI. Noise ceilings were adjusted for test length using the Spearman−Brown correction.

**Extended Data Table 2:**
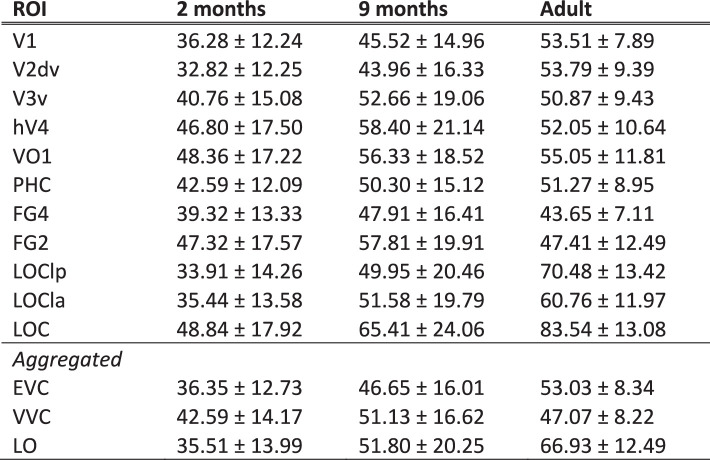
Raw tSNR per ROI/age group

The mean (±s.d.) tSNR value across voxels per ROI.

## Data Availability

Pseudo-anonymized imaging data from infants whose caregivers opted in to public data sharing are available at OpenNeuro (ds006883, 10.18112/openneuro.ds006883.v1.0.0). The RSMs for each ROI and age group presented for analyses in this paper are available as part of the derivatives in the OpenNeuro dataset. For example, derivatives/foundcog_rdms/foundcog-rdms_twomonth_category-level_correlation-distance_julich-rois.pickle contains all RSM combinations for each ROI in the 2-month-old infants. As per our ethical approval, infants who were recruited from the NICU cannot have raw data shared on a publicly available database. The shareable data that are presented in this publication include 135 pictures task fMRI runs from *n* = 78 2-month-old infants and 49 pictures task fMRI runs from *n* = 34 9-month-old infants. Raw BIDS formatted EPI files will be accompanied by events files, preprocessed images after normalization to an age-appropriate template and the FWD values per run. Single-band reference fieldmap scans in opposite phase encoding directions, used for distortion correction, will also be shared. Additionally, we will make available the equivalent data for runs that were not included in the current analysis and the reasons why they were excluded—for example, motion thresholding or manually deemed of poor quality. This brings the total data shared with this release to 173 5-minute awake fMRI runs from 84 2-month-old infants and 64 5-minute awake fMRI runs from 42 9-month-old infants. Further data from a videos task, resting-state fMRI and anatomical scans will be shared alongside upcoming publications. Source data are provided with this paper.

## Code availability

The experimental paradigm is available at https://github.com/ClionaOD/foundcog_paradigm. Preprocessing and analysis pipelines are available at https://github.com/ClionaOD/foundcog_analysis as well as the code used to generate plots from the source data provided.
