# Supplementary material for: Infants have rich visual categories in ventrotemporal cortex at 2 months of age
Source: Nat Neurosci. 2026 Feb 2;29(3):693–702. doi: 10.1038/s41593-025-02187-8 (PMC12971487; doi:10.1038/s41593-025-02187-8)
Supplement: Supplementary file 1 — The consent form used for the study [file 41593_2025_2187_MOESM1_ESM.pdf]

nature neuroscience

Supplementary information

https://doi.org/10.1038/s41593-025-02187-8

Infants have rich visual categories in ventrotemporal cortex at 2 months of age

In the format provided by the authors and unedited

CAREGIVER CONSENT FORM

TITLE OF STUDY: Using Neuroimaging to Measure Functional Brain Development in Infants From the Neonatal Intensive Care Unit and a Control Group

There are 4 sections in this form. Each section contains a number of statements. You are asked to write your initials in the box beside the statement if you agree. If you do not agree with a statement, please leave the box blank. The end of this form is for the researchers to complete.

Please ask the researchers any questions you may have when reading each of the statements.

Thank you for participating.

GENERAL CONSENT	Caregiver Initials
I confirm I have read and understood the Information Leaflet for the above-named study. The information has been fully explained to me and I have been able to ask questions, all of which have been answered to my satisfaction.	
I understand that participation in this study **is entirely voluntary, and if I decide that I do not want to take part, I can stop taking part in this study at any time without giving a reason** ***and I understand that opting out won't affect the future medical care of my child.***	
I know how to contact the research team if I need to.	
I agree to being contacted by researchers by email or phone as part of this research study.	
I agree to take part in this research study having been fully informed of the **risks, benefits and alternatives** which are set out in full in the information leaflet with which I have been provided.	
I consent to take part in this research study with my child having been fully informed of the risks, benefits and alternatives.	
I understand the risks related to the spread of the SARS-CoV-2 virus and I consent to take part in the research study and to follow the safety procedure thoroughly.	

Coláiste na Tríonóide, Baile Átha Cliath
Trinity College Dublin
Ollscoil Átha Cliath | The University of Dublin

**DATA PROCESSING**	**Caregivers Initials**
I give my permission for my and my child's data to be processed in line with the aims of the research study, as outlined in the information leaflet.	
I understand that **results from analysis of my personal and my child's information will not be given to me.**	
I understand that, under the Freedom of Information Act (2014), and the GDPR I can have access to any identifiable information the study team stores about me and my child, if requested.	
I understand that the personal information collected in the study will be kept strictly confidential and will only be made available to qualified scientists who are part of the study team.	
I understand that I can **withdraw my permission to take part in this study at any time** without giving a reason.	
I give permission for researchers to collect information about my and my child's health status that are relevant to the goal of minimizing the SARS-CoV-2 virus spread.	
I give permission for researchers to look at my child's medical records to get information. I have been assured that information about my child will be kept private and confidential.	
I give permission for the radiographer to share the anatomical images with the responsible neonatal radiologist in order to check them for incidental findings.	
I give permission for the radiologist to contact the responsible neonatologist in case of incidental findings.	
I give permission for researchers to collect my demographic information. I have been assured that information about me will be kept private and confidential.	
I understand that the anatomical images necessary to check for incidental findings will only be acquired if my infant falls asleep in the scanner and that the research cannot be expected to give diagnostic information.	

[OPTIONAL] I give permission to use **recognizable video data** of my child in scientific presentations related to this study.	
[OPTIONAL] I give permission to use **recognizable video data** of my child in scientific publications (i.e. including part of the video data in video abstracts in order to make our work clearer and more reproducible by other researchers).	
[OPTIONAL] I give permission to use **recognizable video data** on the lab website for advertising purposes.	
I understand that confidentiality may be breached in circumstances in which;<br>1. The research team has a strong belief or if evidence exists that there is a serious risk of harm or danger to either the participant or another individual. This may relate to issues surrounding physical, emotional and/or sexual abuse, concerns for child protection, rape, self-harm, suicidal intent or criminal activity.<br>2. Disclosure is required as part of a legal process or Garda investigation. In such instances, information may be disclosed to significant others or appropriate third parties without permission being sought. Where possible, a full explanation will be given to the participant regarding the necessary procedures and also the intended actions that may need to be taken.	

Coláiste na Tríonóide, Baile Átha Cliath
Trinity College Dublin
Ollscoil Átha Cliath | The University of Dublin

<table>
<tr><th colspan="2">RETENTION OF DATA FOR FUTURE RESEARCH</th></tr>
<tr><td rowspan="3">I give permission for material/data to be stored for possible future research related to the current study at the time of the future research but only if the research is approved by a Research Ethics Committee.</td><td>Yes, without further consent being required</td></tr>
<tr><td>Yes, but only if additional consent is obtained</td></tr>
<tr><td>No</td></tr>
<tr><td>I understand that pseudo-anonymised data will be retained for a period of 10 years by the study team.</td><td></td></tr>
<tr><td>SHARING OF INFORMATION [OPTIONAL]</td><td>Caregivers Initials</td></tr>
<tr><td>I give permission for my and my child’s pseudo-anonymized data, including the brain imaging data, to be shared with the scientific community and the general public via a fully open database on the internet. <u>I understand that my data will be pseudo-anonymised before sharing - no personally identifying data will be shared</u>. I understand that data shared in this way will be accessible to researchers and members of the public anywhere in the world, not just the EU. I understand that by sharing data in this way, my data might be used for other, future research projects in addition to the study I am currently participating in. Those future projects can focus on any topic and might be completely unrelated to the goals of this study. I understand that once data are shared, <u>they cannot be destroyed, withdrawn, or recalled, because they can no longer be linked with me.</u> I understand that it is possible that some of the research conducted using my shared information eventually could lead to the development of new research methods, new diagnostic tests, new drugs, or other commercial products. I understand that should this occur, there is no plan to provide me, the study team, or TCD with any part of the profits generated from such products, nor will I, the study team, or TCD have any ownership rights in the products</td><td></td></tr>
<tr><td>I give permission to share recognizable video data of my child in a public research repository to which both EU and non-EU researchers will have access. Video data sharing shall be subject to Standard Contractual Clauses being put in place for the use of the video data</td><td></td></tr>
</table>

Coláiste na Tríonóide, Baile Átha Cliath
Trinity College Dublin
Ollscoil Átha Cliath | The University of Dublin

Caregiver Name (Block Capitals) Caregiver Signature
Date:

-- --

Witness Name (Block Capitals) Witness Signature Date

To be completed by the Principal Investigator or nominee:

I, the undersigned, have taken the time to fully explain to the above participant the nature and purpose of this study in a way that they could understand. I have explained the risks and possible benefits involved. I have invited them to ask questions on any aspect of the study that concerned them.

I have given a copy of the information leaflet and consent form to the participant with contacts of the study team

Researcher name:

Title and qualifications:

Signature:

Date:

Copies to be made: 1 for participant, 1 for PI (and in some cases additional copies for hospital records).

Coláiste na Tríonóide, Baile Átha Cliath
Trinity College Dublin
Ollscoil Átha Cliath | The University of Dublin
