# Supplementary material for: Infants have rich visual categories in ventrotemporal cortex at 2 months of age
Source: Nat Neurosci. 2026 Feb 2;29(3):693–702. doi: 10.1038/s41593-025-02187-8 (PMC12971487; doi:10.1038/s41593-025-02187-8)
Supplement: Supplementary file 2 — Reporting Summary [file 41593_2025_2187_MOESM2_ESM.pdf]

nature portfolio

Corresponding author(s): Cliona O'Doherty, PhD

Last updated by author(s): 02/11/2025

Reporting Summary

Nature Portfolio wishes to improve the reproducibility of the work that we publish. This form provides structure for consistency and transparency in reporting. For further information on Nature Portfolio policies, see our Editorial Policies and the Editorial Policy Checklist.

Statistics

For all statistical analyses, confirm that the following items are present in the figure legend, table legend, main text, or Methods section.

n/a	Confirmed	
☐	☒	The exact sample size (*n*) for each experimental group/condition, given as a discrete number and unit of measurement
☐	☒	A statement on whether measurements were taken from distinct samples or whether the same sample was measured repeatedly
☐	☒	The statistical test(s) used AND whether they are one- or two-sided<br>*Only common tests should be described solely by name; describe more complex techniques in the Methods section.*
☐	☒	A description of all covariates tested
☐	☒	A description of any assumptions or corrections, such as tests of normality and adjustment for multiple comparisons
☐	☒	A full description of the statistical parameters including central tendency (e.g. means) or other basic estimates (e.g. regression coefficient) AND variation (e.g. standard deviation) or associated estimates of uncertainty (e.g. confidence intervals)
☐	☒	For null hypothesis testing, the test statistic (e.g. *F*, *t*, *r*) with confidence intervals, effect sizes, degrees of freedom and *P* value noted<br>*Give P values as exact values whenever suitable.*
☒	☐	For Bayesian analysis, information on the choice of priors and Markov chain Monte Carlo settings
☒	☐	For hierarchical and complex designs, identification of the appropriate level for tests and full reporting of outcomes
☒	☐	Estimates of effect sizes (e.g. Cohen's *d*, Pearson's *r*), indicating how they were calculated

Our web collection on statistics for biologists contains articles on many of the points above.

Software and code

Policy information about availability of computer code

Data collection	PsychoPy v2022.1.4<br>Python v3.7.12<br>Paradigm is available at https://github.com/ClionaOD/foundcog_paradigm
Data analysis	Python v3.8.10<br>HeuDiConv v0.10.0<br>NiPype v1.8.5<br>FSL v6.0<br>fMRIPrep v20.2.2<br>ANTS v2.4.4<br>Nilearn v0.9.2<br>Nibabel v4.0.2<br>SciPy v1.10.1<br>PyTorch v2.0.1<br>CUDA 11.7<br>torchvision v0.15.2<br>nltk 3.5<br>gensim 4.2.0<br>Preprocessing and analysis code are available at https://github.com/ClionaOD/foundcog_analysis

For manuscripts utilizing custom algorithms or software that are central to the research but not yet described in published literature, software must be made available to editors and reviewers. We strongly encourage code deposition in a community repository (e.g. GitHub). See the Nature Portfolio guidelines for submitting code & software for further information.

Data

Policy information about availability of data

All manuscripts must include a data availability statement. This statement should provide the following information, where applicable:

- Accession codes, unique identifiers, or web links for publicly available datasets
- A description of any restrictions on data availability
- For clinical datasets or third party data, please ensure that the statement adheres to our policy

Pseudo-anonymized imaging data from infants whose caregivers who opted in to public data sharing are available OpenNeuro (OpenNeuro accession number: ds006883, doi:10.18112/openneuro.ds006883.v1.0.0). The RDMs for each ROI and age group presented for analyses in this paper are available as pickle files as part of the derivatives in the OpenNeuro dataset (e.g., derivatives/foundcog_rdms/foundcog-rdms_twomonth_category-level_correlation-distance_julich-rois.pickle contrain all RDM combinations for each ROI in the 2-month-olds). As per our ethical approval, infants who were recruited from the NICU cannot have raw data shared on a publicly available database. The shareable data that is presented in this publication includes 135 pictures task fMRI runs from n=78 2-month-olds and 49 pictures task fMRI runs from n=34 9-month-olds. Raw BIDS formatted epi files will be accompanied by events files, pre-processed images after normalisation to an age appropriate template, and the framewise displacement values per run. Single band reference fieldmap scans in opposite phase encoding directions, used for distortion correction, will also be shared. Additionally, we will make available the equivalent data for runs that were not included in the current analysis and reasons why they were excluded; for example, motion thresholding or manually deemed of poor quality. This brings the total data shared with this release to 173 5 min awake fMRI runs from 84 2-month-olds, and 64 5 min awake fMRI runs from 42 9-month-olds. The videos task, resting state fMRI and anatomical scans will be shared alongside upcoming publications.

Research involving human participants, their data, or biological material

Policy information about studies with human participants or human data. See also policy information about sex, gender (identity/presentation), and sexual orientation and race, ethnicity and racism.

Reporting on sex and gender

The fMRI dataset consisted of 130 2-month-olds (52 female, 78 male) and 66 9-month-olds (31 female, 35 male). After strict motion correction, the sample included in MVPA was 101 2-month-olds (64 male, 37 female) and 44 9-month-olds (22 male, 22 female).

Reporting on race, ethnicity, or other socially relevant groupings

No race, ethnicity or socially relevant groupings are reported in this manuscript. The socio-economic data for all infants was collected for completeness, but has not yet been used for reporting.

Population characteristics

Infants were recruited from participating maternity hospitals and attended two scanning sessions at Trinity College Institute of Neuroscience (TCIN). The first scan was scheduled for as close as possible to when the infant was 2-months corrected gestational age (CGA) and another at 9-months CGA. 28 participants' caregivers chose not to continue in the study at the 9-month follow up and 3 infants were not invited back due to poor tolerance of the scanning at 2-months. Awake functional MRI was successful in 97% of 2-month-olds and 64% of 9-month-olds. The final dataset consisted of 130 2-month-olds (52 female, 78 male, 1.5 – 4.7 months CGA, mean=2.4 months), 66 9-month-olds (31 female, 35 male, 7.5 – 10.9 months CGA, mean=9.3 months) and was composed of infants born healthy at full-term (n=101 2-months, n=55 9-months) as well as a smaller proportion who were born pre-term and spent time in the neonatal intensive care unit (NICU) (n=29 2-months, n=11 9-months).

Recruitment

Participants in the non-clinical group were recruited through the participating hospitals' maternity wards. Approved researchers consulted with nurses on the ward about the caregivers who could be approached without causing distress. The researcher then approached those caregivers, explained the research and collected their information if they were interested in being contacted at a future date. All researchers explicitly stated that agreeing to be contacted does not include an agreement to participate in the research, and they are free to have their contact details deleted at any time. One month later, caregivers received an email or phone call explaining the details of the study and asked if they would like to participate. The first scan was then scheduled for when the infant was 2-months-old, and consent forms were signed on the day of data collection. Parents gave informed consent on behalf of their infants to participate in the research study after reviewing the relevant participant information leaflet which had been provided prior to the scheduled scan session. Participants in the NICU group were recruited similarly, with the exception of first being approached in the NICU unit of the relevant maternity hospital by a clinical member of the team. There is potential self-selection bias in the NICU cohort, as the caregivers of infants who were doing well after discharge were more likely to participate in the study. This has the potential to bias any future studies that seek to separate the clinical cohort from the healthy group, as those in the clinical subset were more likely to display good developmental outcomes despite pre-term birth. The recruitment protocol was discussed in a pre-award ethical review with the European Union prior to the study being funded, and was approved by TCD, The Rotunda and The Coombe ethics committees.

Ethics oversight

The study was conducted under ethical approval from the Trinity College Dublin School of Psychology Research Ethics Committee (SPREC012020-10), The Rotunda Ethics Committee (REC-2019-018) and The Coombe ethics committee (Study No. 8 – 2019).

Note that full information on the approval of the study protocol must also be provided in the manuscript.

Field-specific reporting

Please select the one below that is the best fit for your research. If you are not sure, read the appropriate sections before making your selection.

- [x] Life sciences
- [ ] Behavioural & social sciences
- [ ] Ecological, evolutionary & environmental sciences

For a reference copy of the document with all sections, see nature.com/documents/nr-reporting-summary-flat.pdf

Life sciences study design

All studies must disclose on these points even when the disclosure is negative.

Sample size	The minimum recommended sample size for fMRI is N=40 usable datasets. We projected to add a conservative margin of 25% (N=50 usabl e datasets) yielding 80% power for a medium-to-large effect size of r=0.4. We conducted modelling to estimate the number of infants that needed to be recruited to achieve this. The typical success rate per session is 55-65% for this age group, regardless of whether scanning is conducted while the infant is awake or asleep, therefore we aimed for a sample of N=166 to be recruited. We made initial contact with 932 caregivers, 170 of whom were interested in participating and 137 progressed to participate scanning. found that our real data collection efforts were far more successful than the projected number at 97%, leading to a final dataset of N=130 usable datasets at 2-months-old and N=66 9-month-olds.This was further reduced to n=101 2-month olds and n=44 9-month-olds included in the results presented in this manuscript.
Data exclusions	Of the 130 2-month-olds and 65 9-month-olds with successful awake data collected, 112 and 52 infants progressed from the videos to the pictures task. Runs that were deemed to be of poor quality by attending researchers were manually excluded from later analyses, resulting in 108 2-month-olds and 48 9-month-olds who progressed to the modelling presented here. A final exclusion came from motion thresholding. Runs with >50% of frames >1.5 mm framewise displacement were excluded, giving a strict final sample of n=101 2-month-olds and n=44 9-month-olds. Results were validated in a subset of infants matched for motion, exclusive of NICU graduates.
Replication	No exact replication of the experiment was performed for this fMRI study. Multiple runs of the fMRI experiments were collected in each participant. While an exact repeat of the experiment would be challenging do to the nature of the infant fMRI dataset, we ensured reproducibility of the analyses by repeating all analyses multiple times. Custom code was tested, reviewed by other team members, and refactored to ensure consistent results.
Randomization	Randomization was not relevant to the study as we were not testing differences between clinical groups. Instead, we measured both cross-sectionally and longitudinally.
Blinding	Blinding was not relevant to the study as we were not testing differences between clinical groups.

Reporting for specific materials, systems and methods

We require information from authors about some types of materials, experimental systems and methods used in many studies. Here, indicate whether each material, system or method listed is relevant to your study. If you are not sure if a list item applies to your research, read the appropriate section before selecting a response.

Materials & experimental systems

n/a	Involved in the study	
☒	☐	Antibodies
☒	☐	Eukaryotic cell lines
☒	☐	Palaeontology and archaeology
☒	☐	Animals and other organisms
☒	☐	Clinical data
☒	☐	Dual use research of concern
☒	☐	Plants

Methods

n/a	Involved in the study	
☒	☐	ChIP-seq
☒	☐	Flow cytometry
☐	☒	MRI-based neuroimaging

Plants

Seed stocks

Report on the source of all seed stocks or other plant material used. If applicable, state the seed stock centre and catalogue number. If plant specimens were collected from the field, describe the collection location, date and sampling procedures.

Novel plant genotypes

Describe the methods by which all novel plant genotypes were produced. This includes those generated by transgenic approaches, gene editing, chemical/radiation-based mutagenesis and hybridization. For transgenic lines, describe the transformation method, the number of independent lines analyzed and the generation upon which experiments were performed. For gene-edited lines, describe the editor used, the endogenous sequence targeted for editing, the targeting guide RNA sequence (if applicable) and how the editor was applied.

Authentication

Describe any authentication procedures for each seed stock used or novel genotype generated. Describe any experiments used to assess the effect of a mutation and, where applicable, how potential secondary effects (e.g. second site T-DNA insertions, mosiacism, off-target gene editing) were examined.

Magnetic resonance imaging

Experimental design

Design type

Event-related task fMRI

Design specifications

Picture stimuli were 6 x 2 pairs of categories, chosen to relate to a particular context and to be something an infant may encounter in the first year of life. These were cat, crab, bird, squirrel, rubber duck, dishware, fence, food, supermarket shelves, shopping cart, towel and tree. A piloting period in adults helped to inform the decision of the final 12 categories tested. Pictures appeared against a black background for 3 s followed by a fixation cross, with the inter-stimulus interval ranging between 3.5 – 4.5 s. Images loomed towards participants, increasing logarithmically, and doubling in size within the presentation window. There were 3 exemplars for each of the 12 categories and each was repeated twice per fMRI run. This gave a final design of 6 contexts x 2 object types x 3 instances x 2 repetitions, totalling 5 min of scanning. During the task, a series of short nursery rhymes played through the infant headphones to maintain engagement. Backgrounds were removed and the images were rescaled so that the largest dimension, either width or height, touched the image border. Then, images were resized and padded to a standard size of 640 x 360 pixels and pre-distorted to ensure they appeared normal when displayed on the scanner's curved surface.

Behavioral performance measures

No behavioural task was performed. Infants' engagement with the task was monitored through a a live-stream of the infant gaze, which could then be used for subsequent coding.

Acquisition

Imaging type(s)

functional, structural

Field strength

3 Tesla

Sequence & imaging parameters

For infants:
MRI data were collected on a Siemens MAGNETOM Prisma 3T scanner. The task-based fMRI sequences used multiband accelerator factor of 4, voxel size 3 x 3 x 3 mm, FOV 192 x 192 mm, phase encoding direction anterior to posterior, repetition time (TR) 610 ms, echo time (TE) 32 ms, flip angle 40 degrees, echo spacing 0.54 ms, 36 slices per volume. 510 volumes were collected for the pictures task. Two spin echo-planar images in opposite phase encoding directions were collected, with a TR of 2260 ms, a TE of 32 ms, field of view 192 x 192 mm, flip angle 40 degrees, 36 slices per volume, with a total of 10 volumes acquired for a scanning time of 29 s. The two reference scans were used to apply susceptibility distortion correction using FSL topup. T2 weighted images were a noise-reduced scan consisting of the following parameters: 100 contiguous near-axial slices, GRAPPA acceleration factor of 3, voxel size 1 mm × 1 mm x 1 mm, coverage of the whole head and a field of view 192 mm, TR 6090 ms, TE 84 ms, flip angle 150 degrees, echo spacing 12 ms, and duration 4 m and 5 s. Later in the study, a T1 MPRAGE sequence was added with 144 sagittal slices, voxel size 1 mm × 1 mm x 1 mm, coverage of the whole head and a field of view 256 mm, TR 2300 ms, TE 2.98 ms, TI 900 ms, flip angle 9 degrees, echo spacing 7.1 ms, and duration 6 m and 28 s.

For adults:
Adult MRI data were collected using the full 64 Channel Siemens Head Neck coil. The acquisition parameters were as follows: a multiband acceleration factor of 4 was used with a voxel size of 3 x 3 x 3 mm and a FOV of 224 x 245; the phase encoding direction was left to right; repetition time was 656 ms and echo time was 30 ms; flip angle 50 degrees and echo spacing of 0.54 ms. 40 slices were collected per volume and 561 volumes were taken due to the longer design for this piloting study.

Area of acquisition

Wholebrain

Diffusion MRI ☐ Used ☒ Not used

Preprocessing

Preprocessing software

For infants:
MRI data were pre-processed using an in-house pipeline written in Python with the NiPype processing framework to avail of

the neuroimaging software packages FSL and ANTs.

For adults:
The adult dataset was pre-processed using fMRIPrep with FSL Topup.

Normalization	Brain images were co-registered to the mean of a chosen reference scan using an affine transform with FSL FLIRT. Due to limited movement in the infants when they fell asleep, resting state scans were the preferred choice for the functional reference. In the absence of a resting state scan, awake fMRI runs were used instead. The degrees of freedom (DOF) used for the affine transform were chosen by manual inspection for each infant, with the vast majority of participants having a successful registration with DOF=12. Scans in native space were then normalised to a common space with FSL FLIRT.
Normalization template	An age-appropriate NIHPD template was used (Fonov et al., 2009 https://dx.doi.org/10.1016/S1053-81190970884-5). At 2-months-old this was the 02-05 month template, and at 9-months-old the 08-11 month template.
Noise and artifact removal	Two fieldmap reference scans with opposite phase encoding (A/P and P/A) were input to FSL topup to estimate the susceptibility-induced off-resonance field. EPI images were motion corrected to a middle reference volume using FSL MCFLIRT and converted to SPM format from which framewise displacement and DVARS were calculated. The field distortion estimation from topup was then applied to the EPI data to correct for susceptibility distortions. Three translation and three rotation motion regressors calculated during pre-processing were included as covariates in the generalised linear model, as well as a linear trend regressor and a cosine drift model with a high pass filter of 0.01 Hz.
Volume censoring	The framewise displacement values calculated during pre-processing were used to apply censoring in frames that exceeded a threshold of 1.5 mm, thereby removing these frames from parameter estimation. If greater than 50% of the frames in a run were above this motion threshold, it was discarded from further analyses.

Statistical modeling & inference

Model type and settings	A volumetric whole-brain general linear model (GLM) was fit for each functional run with custom Python scripts using the Nilearn package (v0.9.2). A design matrix was constructed with 36 regressors of interest, one for each object/exemplar, convolved with a canonical Glover HRF function. For a subset of the analyses, we used a separate model in which conditions were collapsed across exemplars giving 12 regressors of interest, but all other model details were kept the same.
Effect(s) tested	No univariate contrasts or statistics are reported in this manuscript as we focus instead on multivariate approaches. Betas were estimated for each category in all voxels.

Specify type of analysis: ☐ Whole brain ☒ ROI-based ☐ Both

Anatomical location(s)	The cytoarchitectonic Julich atlas70 was transformed into the infant space to define various ROIs. Regions in this atlas have previously been validated to overlap with functionally defined retinotopic areas71,72, enabling us to select a subset of visual ROIs. These were V1/hOc1, V2dv/hOc2, V3v/hOc3v, hV4/hOc4v, VO1/FG1 and PHC/FG3, all referred to in-text by their functional name. Additional cytoarchitecturally defined regions were FG2 and FG4 on the fusiform gyrus, and lateral object selective regions LOC/hOc5, LOCla/hOc4la and LOClp/hOc4lp which were validated to overlap with object regions using neurosynth.org. Aggregated ROIs were used to provide broadly interpretable measures along the ventral stream. These were EVC (V1+V2dv+V3v), VVC (VO1+PHC+FG2+FG4) and LO (LOC+LOCla+LOClp).

Statistic type for inference<br>(See Eklund et al. 2016)	Statistics were performed using non-parametric bootstrap resampling across subjects to estimate the confidence intervals for various tests of correlation. No univariate contrasts or statistics are reported in this manuscript as we focus instead on multivariate approaches.
Correction	We did not correct for multiple comparisons as no univariate contrasts or statistics are reported in this manuscript.

Models & analysis

n/a	Involved in the study
☒	☐ Functional and/or effective connectivity
☒	☐ Graph analysis
☐	☒ Multivariate modeling or predictive analysis

Multivariate modeling and predictive analysis	The run-wise parameter estimates (betas) for each condition within each ROI were calculated as well as the variance/covariance (vcov) matrix across timepoints of the design, multiplied by the residual mean square image to estimate dispersion. To control for differences in baseline signal within each fMRI run, we performed run-level mean centring of estimates by subtracting each voxel's run-level mean across all trials from all estimates within a run, as has been shown to improve MVPA results82. Due to the high motion in some infant runs and the use of censoring, a small percentage of model fits resulted in unstable parameter estimates and very noisy betas. To overcome this, the vcov estimates were used to threshold the voxelwise betas. If a particular voxel had a vcov dispersion >10 it was excluded from the MVPA, this value was chosen by cross-validation of a range of thresholds and their effect on the resulting beta distribution. The proportion of voxels excluded from the aggregate ROIs in each age group were as follows. Average proportion excluded in 2-month-olds: EVC median=3.79% (IQR 0.76% – 9.56%); VVC 3.47% (0.77% - 8.43%) and LO 1.7% (0.24% - 8.76%). In 9-month-olds this was EVC 1.64% (0.72% - 4.88%); VVC 2.25% (0.26% - 8.18%) and LO 0.39% (0.00% - 2.06%). Adult EVC 7.29% (5.12% - 8.66%); VVC 8.98% (4.64%, 13.73%) and LO (2.66% (1.35%, 4.05%).

The pairwise correlations between the 36 objects' voxelwise response patterns were calculated across all unique pairs of subjects and runs (2-months: 14108 unique subject/run pairs, 9-months: 1995 pairs, adults: 136 pairs). These across subject-pair RDMs were then averaged to calculate a group visual representation, ensuring that observed patterns were restricted to signals that were common across the group. Distances were centred about 0 which resulted in highly consistent group average correlations, but small magnitudes that varied in scale with age [2-months (-0.027, 0.030); 9-months (-0.053, 0.073); adults (-0.11, 0.14)]. Therefore, we z-scored when plotting the RDMs to focus on representational content, in terms of relational geometry, rather than strength.
